# A kidney resident macrophage subset is a candidate biomarker for renal cystic disease in preclinical models

**DOI:** 10.1242/dmm.049810

**Published:** 2023-01-16

**Authors:** Zhang Li, Kurt A. Zimmerman, Sreelakshmi Cherakara, Phillip H. Chumley, James F. Collawn, Jun Wang, Courtney J. Haycraft, Cheng J. Song, Teresa Chacana, Reagan S. Andersen, Mandy J. Croyle, Ernald J. Aloria, Raksha P. Hombal, Isis N. Thomas, Hanan Chweih, Kristin L. Simanyi, James F. George, John M. Parant, Michal Mrug, Bradley K. Yoder

**Affiliations:** ^1^ Department of Cell, Developmental, and Integrative Biology, University of Alabama at Birmingham, Birmingham, AL 35294, USA; ^2^ Division of Nephrology, Department of Internal Medicine, University of Oklahoma Health Sciences Center, Oklahoma City, OK 732104, USA; ^3^ Department of Medicine, University of Alabama at Birmingham, Birmingham, AL 35294, USA; ^4^ Department of Veterans Affairs Medical Center, University of Alabama at Birmingham, Birmingham, AL 35233, USA; ^5^ Department of Pharmacology and Toxicology, University of Alabama at Birmingham, Birmingham, AL 35294, USA; ^6^ Department of Surgery, University of Alabama at Birmingham, Birmingham, AL 35294, USA

**Keywords:** ADPKD, Biomarker, CD206, Renal disease and progression, Renal macrophages

## Abstract

Although renal macrophages have been shown to contribute to cyst development in polycystic kidney disease (PKD) animal models, it remains unclear whether there is a specific macrophage subpopulation involved. Here, we analyzed changes in macrophage populations during renal maturation in association with cystogenesis rates in conditional *Pkd2* mutant mice. We observed that CD206^+^ resident macrophages were minimal in a normal adult kidney but accumulated in cystic areas in adult-induced *Pkd2* mutants. Using *Cx3cr1* null mice, we reduced macrophage number, including CD206^+^ macrophages, and showed that this significantly reduced cyst severity in adult-induced *Pkd2* mutant kidneys. We also found that the number of CD206^+^ resident macrophage-like cells increased in kidneys and in the urine from autosomal-dominant PKD (ADPKD) patients relative to the rate of renal functional decline. These data indicate a direct correlation between CD206^+^ resident macrophages and cyst formation, and reveal that the CD206^+^ resident macrophages in urine could serve as a biomarker for renal cystic disease activity in preclinical models and ADPKD patients.

This article has an associated First Person interview with the first author of the paper.

## Teaser

**Summary:** This study reveals that CD206^+^ kidney resident macrophages are directly associated with periods of rapid cyst formation and could serve as a biomarker for renal cystic disease in autosomal-dominant polycystic kidney disease patients.

## INTRODUCTION

Polycystic kidney diseases (PKDs) affect more than 500,000 people in the United States and 13 million people worldwide (Paul and Vanden Heuvel, 2014). Among them, the most prevalent is autosomal-dominant PKD [ADPKD; Online Mendelian Inheritance in Man (OMIM) 173900 and 613095]. The vast majority of ADPKD patients carry mutations in *PKD1* or *PKD2*, genes that encode polycystin 1 (PC1) and polycystin 2 (PC2).

The developmental state significantly influences the severity of cystic diseases. In ADPKD, renal cysts detected *in utero* grow much faster than the cyst volume expansion in adult kidneys (Grantham et al., 2010). Similarly, in orthologous PKD mouse models, the rate of cystogenesis is influenced by the age at which the polycystin proteins are inactivated. Cyst growth occurs faster when polycystins are lost in juvenile mice than when the same proteins are lost in adult mice (Piontek et al., 2007). The mechanisms driving these differential rates of cystogenesis are unknown (Piontek et al., 2007; Davenport et al., 2007; Lantinga-van Leeuwen et al., 2007).

Several lines of evidence suggest that injury and inflammatory cells, such as macrophages, are involved in cyst development. Injury accelerates rates of cyst formation in adult-induced cystic mouse models (Zimmerman et al., 2019b; Patel et al., 2008; Takakura et al., 2009; Happe et al., 2009; Bell et al., 2011), and the number of macrophages is increased in kidneys of PKD patients and orthologous animal models (Zeier et al., 1992; Mrug et al., 2013; Karihaloo, 2015; Li et al., 2021). Genetic or pharmacological approaches have been performed to reduce the number or prevent the accumulation of macrophages in preclinical animal models; however, some studies showed beneficial effects by reducing cyst formation and rescuing renal function (Swenson-Fields et al., 2013; Karihaloo et al., 2011), whereas others did not (Zoja et al., 2015; Salah et al., 2019). Thus, the role of macrophages in cyst formation and growth remains unclear.

In mice, renal macrophages can be categorized into two populations based on their origin (Munro and Hughes, 2017; Liu et al., 2020; Hoeffel et al., 2015; Gomez Perdiguero et al., 2015; Zimmerman et al., 2019a). Infiltrating macrophages (herein also referred to as R1 macrophages and defined as F4/80^low^; CD11b^high^) are derived from the bone marrow and are recruited into tissues following injury or infection. Resident macrophages (herein also referred to as R2 macrophages and defined as F4/80^high^; CD11b^low^) are initially derived from embryonic progenitors in the yolk sac and fetal liver and migrate into the kidneys during organogenesis, where they proliferate *in situ* throughout adulthood (Li et al., 2021; Munro and Hughes, 2017). Recent evidence, however, indicates that monocytes from bone marrow can contribute to the R2 population with age, but it is uncertain whether these bone marrow-derived resident-like macrophages are functionally equivalent (Liu et al., 2020). Moreover, the R2 population in the kidney undergoes transcriptional changes from juvenile to adult stages (Lever et al., 2019). Our previous studies demonstrated that renal resident macrophages promote cyst growth in the *Ift88* mutant kidney after ischemia/reperfusion injury and their depletion reduces cyst severity (Zimmerman et al., 2019b). Additionally, resident macrophages in adult kidneys resemble the juvenile kidney R2 profiles following injury (Lever et al., 2019). Thus, resident macrophages with a juvenile-like transcriptional profile are associated with periods of rapid cyst formation.

Here, we analyzed infiltrating (R1) macrophage and resident (R2) macrophage populations during renal maturation in the mouse and evaluated their importance in an orthologous ADPKD mouse model of cystic kidney disease (*Pkd2* mutant) in the absence of renal injury. Our data indicate that a subset of resident macrophages with CD206 expression (CD206^+^ R2) is present during juvenile periods but declines during kidney maturation. This change correlates with the transition from rapid to slow cyst formation in the juvenile- and adult-induced *Pkd2* mutant models. Reducing renal macrophages using *Cx3cr1*-deficient mice attenuated cyst severity in adult-induced *Pkd2* mutants in the absence of injury. Our previous single-cell RNA-sequencing (scRNA-seq) data indicated that the CD206^+^ R2 population is also present in human kidneys (Chung et al., 2020), and here we find that the number of CD206^+^ R2 cells is significantly increased in kidney and urine samples from ADPKD patients. This suggests that the CD206^+^ macrophage-like cells in the urine could serve as a non-invasive biomarker of cystic disease activity.

## RESULTS

### Renal resident macrophages undergo a phenotypic switch during postnatal kidney maturation

**Fig. 1.**
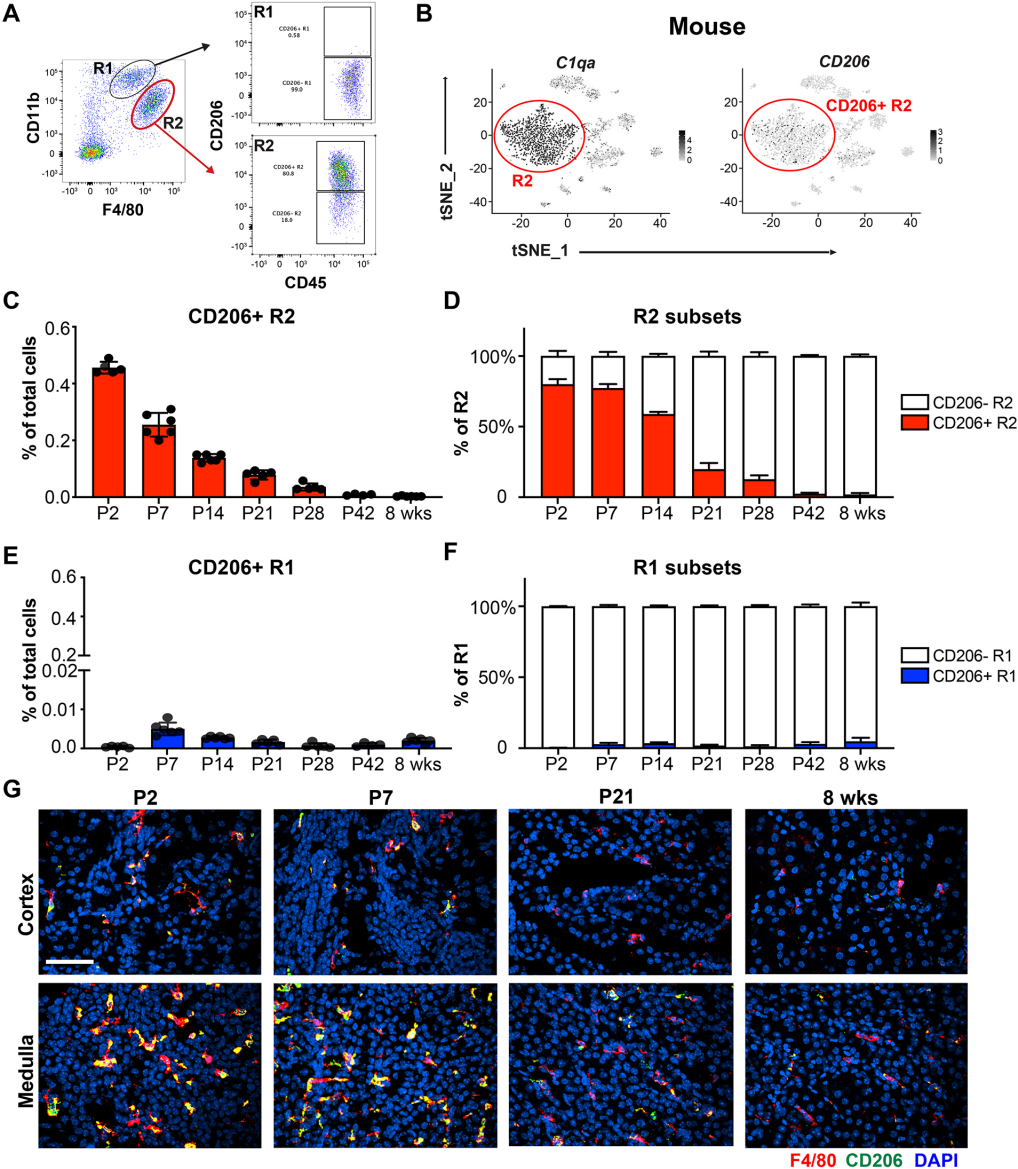
**Renal resident macrophages undergo a phenotypic switch during postnatal kidney maturation.** (A) Flow cytometry gating strategy for infiltrating (R1) and resident (R2) macrophages and the sub-populations expressing CD206. (B) t-distributed stochastic neighbor embedding (t-SNE) plots of single-cell RNA-sequencing (scRNA-seq) data of innate immune cells from mouse kidneys. t-SNE plots depict genes used to identify resident macrophages (*C1qa*) and CD206^+^ resident macrophages (*CD206*). (C,D) Bar graphs showing the percentages of CD206^+^ R2 of total renal cells (C) and of R2 sub-populations (CD206^−^ versus CD206^+^) (D) analyzed in the kidney at postnatal day (P)2, P7, P14, P21, P28, P42 and 8 weeks (wks). (E,F) Bar graphs showing the percentages of CD206^+^ R1 of total renal cells (E) and of R1 sub-populations (CD206^−^ versus CD206^+^) (F) at the indicated time points with flow cytometry analysis. Error bars represent ±s.d. *n*≥5 for each group. (G) Representative confocal images showing F4/80 (red), CD206 (green) and 4′,6-diamidino-2-phenylindole (DAPI; blue) staining in the cortex and medulla of wild-type (WT) kidneys harvested at the indicated time points. Scale bar: 50 μm.

There are different rates of cystogenesis in juvenile versus adult kidneys of PKD animal models (Piontek et al., 2007; Davenport et al., 2007; Lantinga-van Leeuwen, 2007). To test whether changes in macrophages populations correlate with the differential rate of cyst formation, we characterized the number, subtypes and localization of renal macrophages during development in wild-type (WT) mouse kidneys from postnatal day (P)2 to 8 weeks (adult). Infiltrating and resident macrophages were gated as described previously based on the differential expression of CD11b (also known as ITGAM) and F4/80 (also known as ADGRE1) (Fig. 1A; Fig. S1A) (Zimmerman et al., 2019b; Hoeffel et al., 2015; Gomez Perdiguero et al., 2015). We observed that the resident macrophages were the major population of macrophages found in juvenile kidneys. Their percentage was highest during the juvenile period (P2-P7) and then decreased by P14, and remained stable at ∼0.25% of total cells in the kidney of adult mice (Fig. S1B). scRNA-seq analysis from our previous studies indicated that *CD206* (also known as *Mrc1*, encoding protein CD206) was present in a subpopulation of mouse resident macrophages (Fig. 1B) (Zimmerman et al., 2019a). Additionally, these CD206-expressing macrophages localize in cystic regions in injured cilia mutant mice, but not around non-cystic tubules (Zimmerman et al., 2019b). Thus, we analyzed CD206^+^ resident macrophages as a potential marker associated with macrophages that promote cyst formation. Our data indicate that most resident macrophages express CD206 up until ∼P14 (Fig. 1C,D). After P14, there is a shift from CD206^+^ to CD206^−^, with very few CD206^+^ R2 macrophages present in the normal adult mouse kidney. Although CD206 expression was detected in a very small percentage of infiltrating macrophages, we did not observe a similar change in CD206 expression during renal maturation as observed in the resident population (Fig. 1E,F). Immunofluorescence (IF) microscopy analysis of WT kidneys confirmed the changes in CD206-expressing macrophages quantified via flow cytometry (Fig. 1G). In WT kidney, the majority of CD206^+^ macrophages accumulated in the medullary region. Overall, these data indicate that kidney resident macrophages experience a subtype switch in CD206 expression during early postnatal development that coincides with the differential rate of cyst growth observed following induction of cilia dysfunction (Piontek et al., 2007; Lantinga-van Leeuwen et al., 2007; Sharma et al., 2013).

### Rapid cyst formation occurs in juvenile-induced *Pkd2* mutant kidneys

**Fig. 2.**
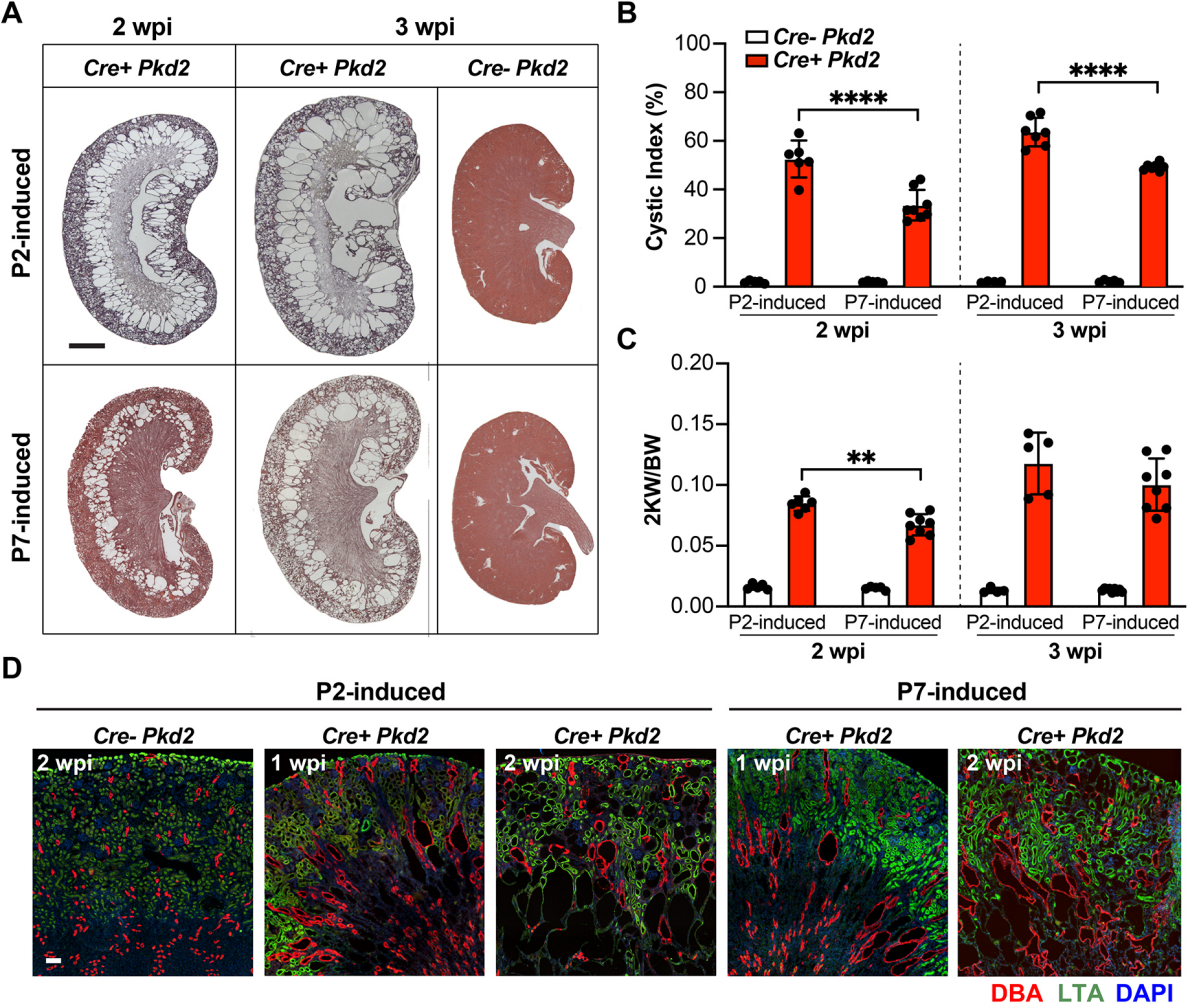
**Rapid cyst formation in juvenile-induced *Pkd2* mutant mouse models.** (A) Representative images of Hematoxylin and Eosin (H&E)-stained kidney sections from *Cre^+^ Pkd2* and *Cre^−^ Pkd2* mice. Samples from P2 (top row) and P7 induction (bottom row) at 2 and 3 weeks post induction (wpi) are shown. Scale bar: 1 mm. (B) The quantification of cystic index in *Cre^−^ Pkd2* and *Cre^+^ Pkd2* mice with P2 and P7 induction analyzed at 2 and 3 wpi. (C) The ratio of the weight of two kidneys to body weight (2KW/BW) in *Cre^−^ Pkd2* and *Cre^+^ Pkd2* at the indicated time points. Each dot represents an individual mouse. Error bars represent ±s.d. *n*≥5 for each group. **P<0.01 and ****P<0.0001 by unpaired two-tailed Student's *t*-test. (D) Representative confocal images showing DBA (red), LTA (green) and DAPI (blue) staining from each group at the indicated time points. Scale bar: 100 μm.

To study the correlation between CD206^+^ resident macrophage accumulation at early postnatal time points and the rapid cyst formation observed in juvenile-induced cystic kidney models, we utilized a conditional *Pkd2* model (*CAG-Cre^ERT2^ Pkd2^fl/fl^*, referred to as *Cre^+^ Pkd2* mice or *Pkd2* mutant mice) and induced the loss of *Pkd2* at two different juvenile time points: P2 and P7. Although rapid cyst formation occurred in both juvenile-induced *Pkd2* mutant mice, there was a more aggressive and severe cystic phenotype in P2-induced *Pkd2* mutant kidneys than in P7-induced *Pkd2* mutant kidneys (Fig. 2A-C). Previous studies have demonstrated that induction of PKD mutation at time points after P14 results in a slow-progressing and more focal cystic kidney phenotype (Piontek et al., 2007). These data indicate that more severe cystic phenotypes corresponded to the higher number of CD206^+^ resident macrophages (Fig. 1D). The most severe cysts in either the P2- or P7-induced mutants were found in the corticomedullary junction despite the Cre recombinase activity throughout the nephron after tamoxifen induction (Fig. S2). IF staining showed that cysts were *Dolichos biflorus* agglutinin (DBA)^+^ or *Lotus tetragonolobus* lectin (LTA)^+^, suggesting they were derived from either proximal tubules or collecting ducts (Fig. 2D).

### CD206^+^ resident macrophages remain present in cystic kidneys following juvenile induction

**Fig. 3.**
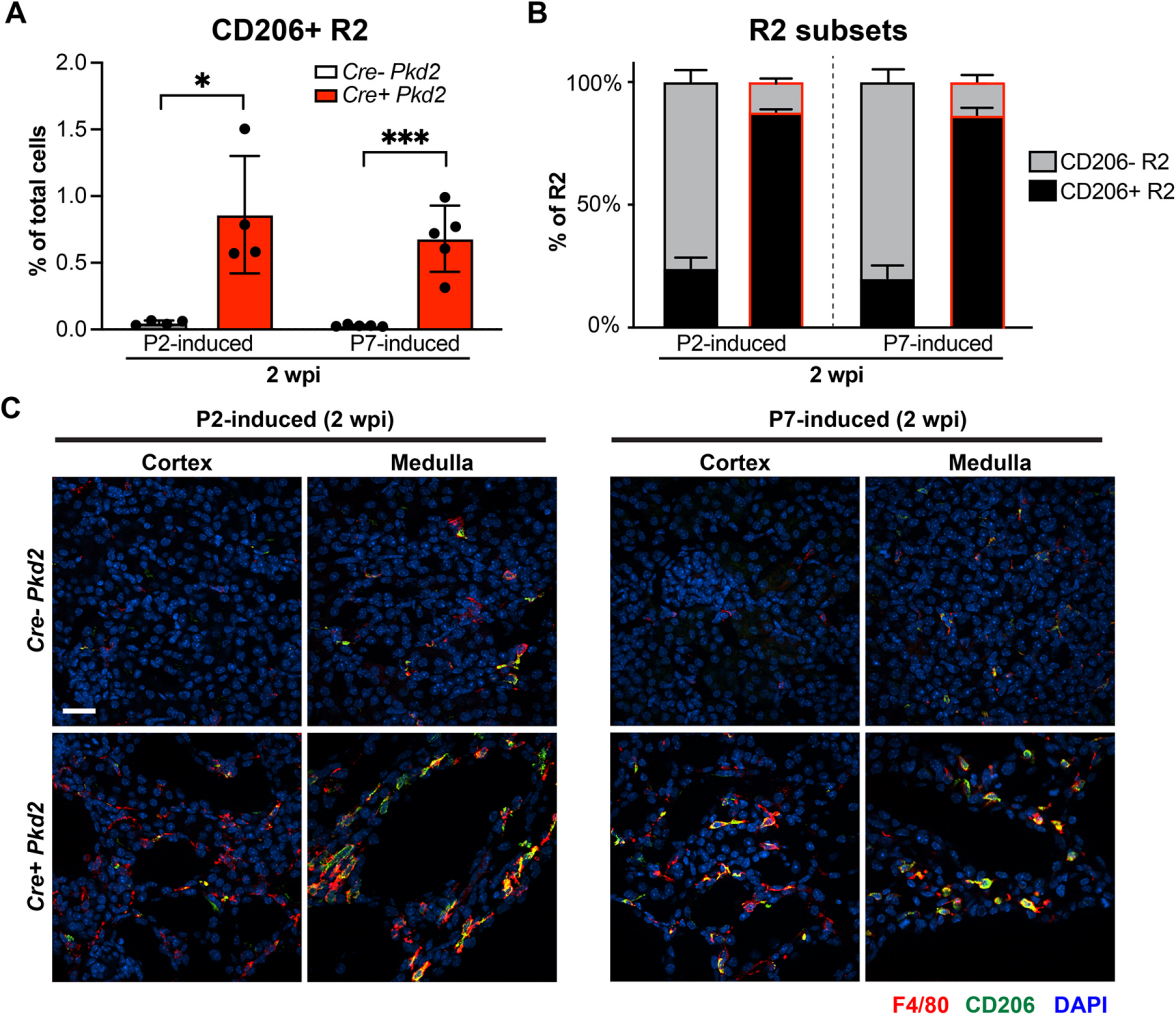
**Accumulated CD206^+^ resident macrophages associated with rapid cyst formation in juvenile-induced *Pkd2* mutant kidneys.** (A) Flow cytometry analysis of the percentage of CD206^+^ R2 macrophages of total renal cells from P2- and P7-induced kidneys at 2 wpi. Each dot represents an individual mouse. Error bars represent ±s.d. *P<0.05 and ***P<0.001 by unpaired two-tailed Student's *t*-test. (B) The percentage of CD206^−^ and CD206^+^ R2 macrophages of total resident macrophages from P2- and P7-induced kidneys analyzed at 2 wpi. The black outline indicates the *Cre^−^ Pkd2* group; the red outline indicates the *Cre^+^ Pkd2* group. Error bars represent ±s.d. *n*≥4 for each group. (C) Representative confocal images showing F4/80 (red), CD206 (green) and DAPI (blue) staining in cortex and medulla from *Cre^−^ Pkd2* and *Cre^+^ Pkd2* mice with P2 (left panel) and P7 induction (right panel) analyzed at 2 wpi. Scale bar: 20 μm.

We investigated the number and percentage of macrophage subtypes 2 weeks post tamoxifen induction (wpi) in the P2- and P7-induced *Pkd2* mutant mice. During renal maturation in control kidney, the level of CD206^+^ resident macrophages (CD206^+^ R2) was low at 2 wpi. In contrast, in the *Pkd2* mutants, the number and percentage of CD206^+^ R2 macrophages were greatly elevated at 2 wpi in both P2- and P7-induced mutants compared to controls (Fig. 3A,B; Fig. S3B). The increase in the percentage of CD206^+^ R2 of total cells and as a percentage of the R2 population paralleled that observed in the juvenile age mice (e.g. P2 or P7). This was not due to the tamoxifen injection as the WT controls did not show this increase. Most of the CD206^+^ macrophages were in regions in which the cysts were located (Fig. 3C; Fig. S3D). It is worth noting that the number of infiltrating macrophages was also increased at 2 wpi in both P2- and P7-induced mutants compared to WT controls, but the dramatic change in CD206 expression that correlated with cyst development was not observed in the infiltrating population (Fig. S3A,C). These data indicate that loss of *Pkd2* disrupts the normal transition of resident macrophages from CD206^+^ to CD206^−^ during kidney maturation and that the CD206^+^ R2 macrophage accumulation occurs in regions with rapid cyst formation.

### Slow focal cyst progression occurs in adult-induced *Pkd2* mutant mice

**Fig. 4.**
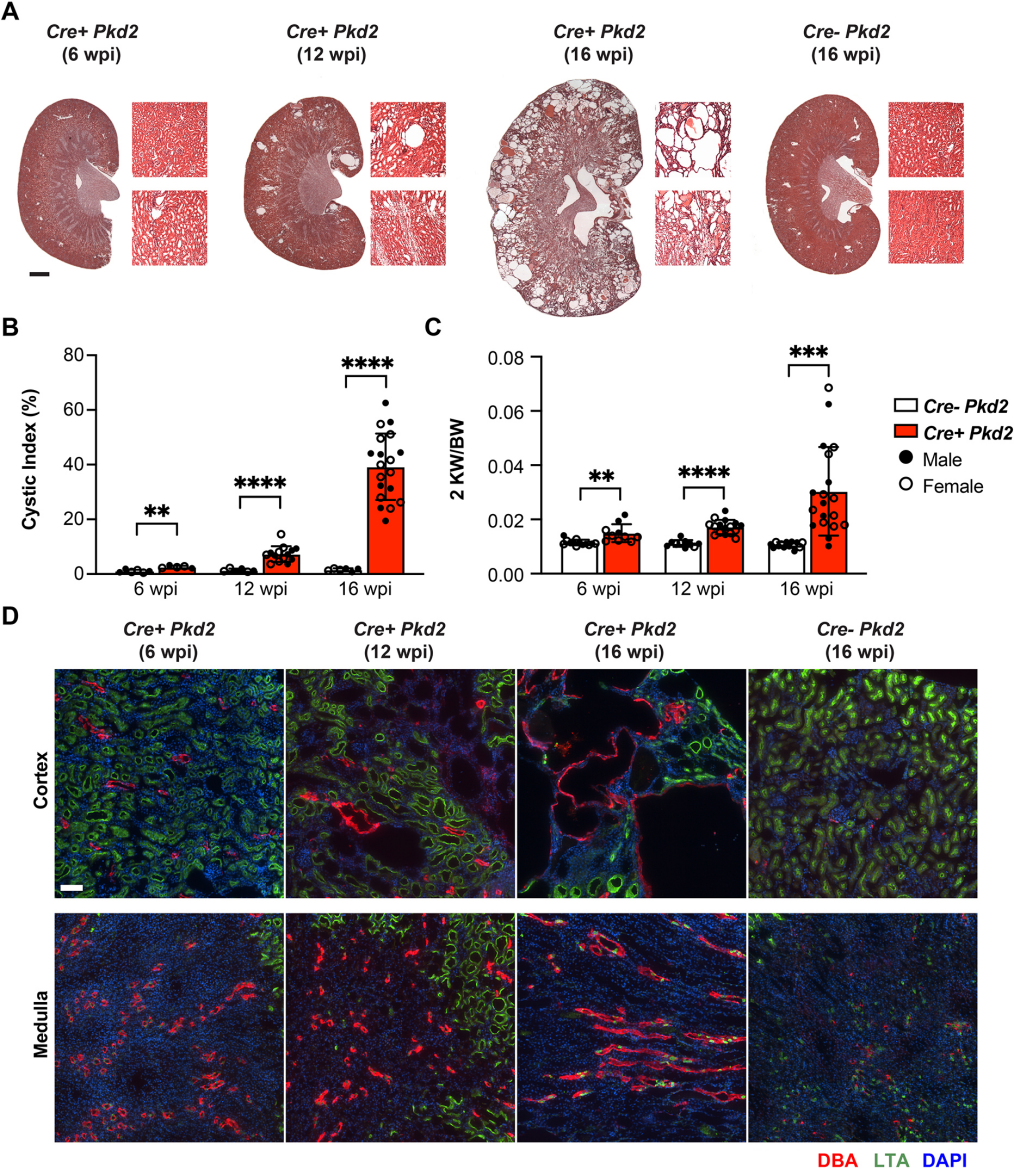
**Slow cyst progression in adult-induced *Pkd2* mutant kidneys.** (A) Representative H&E-stained kidney sections and 10× magnification images for cortex and medulla from *Cre^−^ Pkd2* and *Cre^+^ Pkd2* adult mice induced at the age of 8 weeks. Samples were harvested and analyzed at 6, 12 and 16 weeks post induction (wpi). Scale bar: 1 mm. (B,C) Bar graphs showing the quantification of cystic index (B) and 2KW/BW (C) in *Cre^−^ Pkd2* and *Cre^+^ Pkd2* at the indicated time points. Each dot represents an individual mouse; filled circles and open circles represent male and female mice, respectively. Error bars represent ±s.d. *n*≥10 for each group. **P<0.01, ***P<0.001 and ****P<0.0001 by unpaired two-tailed Student's *t*-test. (D) Representative confocal images showing DBA (red), LTA (green) and DAPI (blue) staining for each group at different time points. Scale bar: 100 μm.

We next investigated the rate of cyst progression and the number of CD206^+^ R2 macrophages in adult-induced *Pkd2* mutant mice. *Pkd2* was disrupted in mice at 8 weeks of age (adult-induced model), and cyst formation and progression were examined at 6, 12 and 16 wpi. In contrast to the rapid cyst formation that occurs in the P2- or P7-induced *Pkd2* mutants, only tubule dilations were observed at 6 wpi in adult-induced *Pkd2* mutant kidneys (Fig. 4A). Although no cysts were evident, the dilations led to an increase in the cystic index and the ratio of the weight of two kidneys to body weight (2KW/BW) in *Pkd2* mutant kidneys at 6 wpi compared to that of the control and became more dramatic at the later time points analyzed (Fig. 4B,C)**.** There was no significant difference in body weight between control and *Pkd2* mutant mice after tamoxifen induction until 16 wpi (Fig. S4). As reported previously, we observed variability in cystic phenotypes despite the full penetration in this PKD mouse model (Dong et al., 2021). We did not detect sex dimorphism regarding cyst severity.

We also found that cysts initiated in a focal manner at 12 wpi and expanded rapidly between 12 and 16 wpi (Fig. 4A). The rapid progression of cyst formation after 12 wpi may be explained by the ‘snowball effect’ originally described by Leonhard et al. (2015), in which the initial focal cyst formation triggers the accelerated progression of PKD in nearby nephrons. The IF microscopy analysis showed that, at 12 wpi, there were dilations/small cysts predominantly in LTA^+^ proximal tubule segments. In contrast, at 16 wpi, most cysts stained positive for DBA, indicating that they were derived from distal collecting tubules (Fig. 4D) and were significantly expanded compared to the LTA dilations/cysts. This suggests two stages of cyst formation in adult-induced *Pkd2* mutant kidneys: a slow focal cyst initiation stage involving proximal tubules, followed by a more rapid cyst progression phase involving DBA^+^ tubules. Overall, this rate of cystogenesis in adult-induced mutant kidneys is much slower than that in juvenile-induced mutant kidneys.

### CD206^+^ resident macrophages accumulate during cyst formation in *Pkd2* mutant kidneys following adult induction

**Fig. 5.**
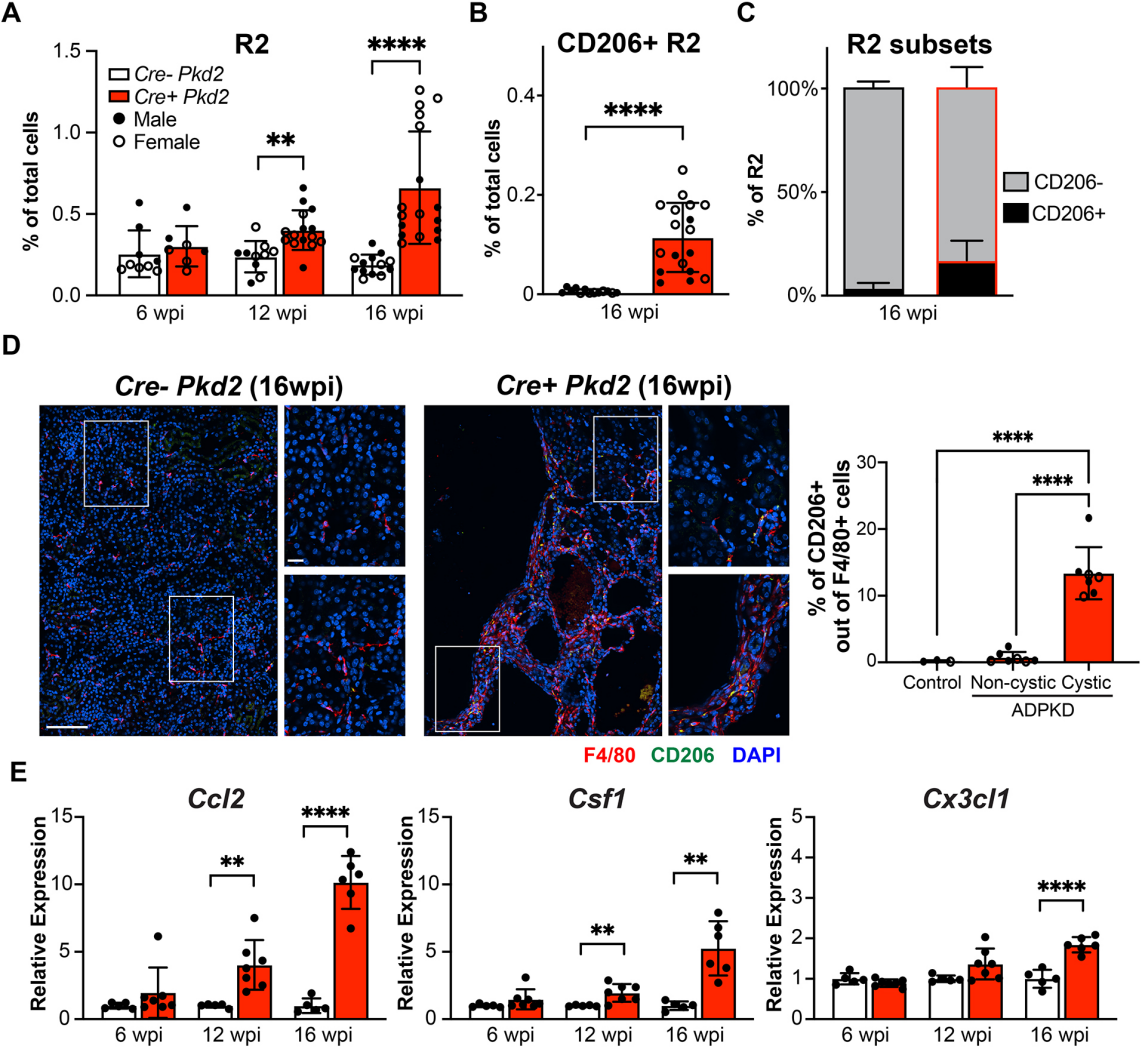
**Accumulated CD206^+^ resident macrophages occur after cyst formation in adult-induced *Pkd2* mutant kidneys.** (A) Bar graph showing the percentage of R2 macrophages of total renal cells in *Cre^−^ Pkd2* and *Cre^+^ Pkd2* at 6, 12 and 16 wpi. (B,C) Bar graphs showing the percentage of CD206^+^ R2 of total cells (B), and the percentage of CD206^+^ cells of R2 macrophages (C), in *Cre^−^ Pkd2* and *Cre^+^ Pkd2* at 16 wpi. Each dot represents an individual mouse; filled circles and open circles represent male and female mice, respectively. Black outline represents *Cre^−^ Pkd2* control samples; red outline represents *Cre^+^ Pkd2* samples. (D) (Left) Representative confocal images showing F4/80 (red), CD206 (green) and DAPI (blue) staining in cortex and medulla in *Cre^−^ Pkd2* and *Cre^+^ Pkd2* at 16 wpi. Scale bar: 100 μm. Insets show representative areas used to analyze cystic and non-cystic regions from *Pkd2* mutant kidney at 16 wpi. Scale bar: 20 μm. (Right) Bar graph showing the percentage of CD206^+^ of F4/80^+^ cells from immunofluorescence staining in non-cystic and cystic areas as represented by the inset boxes. (E) The relative mRNA expression of *Ccl2*, *Csf1* and *Cx3cl1* from whole-kidney lysates of *Cre^−^ Pkd2* and *Cre^+^ Pkd2* at the indicated time points. Each dot represents an individual mouse. Error bars represent ±s.d. *n*≥6 for each group. **P<0.01 and ****P<0.0001 by unpaired two-tailed Student's *t*-test.

The direct correlation between CD206^+^ resident macrophages and cysts in the juvenile-induced model led us to assess whether these macrophages reappear during cyst formation in the adult-induced model. We found no significant increase in R1 and R2 macrophages in *Pkd2* mutant kidneys at 6 wpi related to mild tubule dilation. However, there was a significant increase in the numbers of R1 and R2 macrophages observed at 12 and 16 wpi, correlating with the expanding cysts in the DBA^+^ tubules (Fig. 5A; Fig. S5A). To determine whether CD206^+^ R2 macrophages are associated with the rapid stage of cyst formation in adult-induced *Pkd2* mutants, we analyzed CD206^+^ R2 with flow cytometry and IF microscopy at 16 wpi. The percentage and number of CD206^+^ resident macrophages were significantly increased in *Pkd2* mutant kidneys compared to control kidney, in which CD206^+^ resident macrophages were nearly undetectable (Fig. 5B,C). However, the number of CD206^+^ R2 macrophages and their percentage never approached that observed in the juvenile-induced model. In contrast, there were very few infiltrating R1 macrophages with CD206 expression (less than 0.04% of the total cells), and the percentages of CD206^+^ cells within the R1 subpopulation were less than 2% of those in control or *Pkd2* mutant kidneys at 16 wpi (Fig. S5B,C). Using IF microscopy analysis, there was no overt increase in macrophages in the kidney evident at 6 wpi, even around dilated tubules. In contrast, CD206^+^ macrophage accumulation was evident around cystic areas at 12 wpi in *Pkd2* mutant kidneys (Fig. S5D) and was much more frequent in severe cystic kidneys at 16 wpi (Fig. 5D).

To evaluate the possible mechanisms driving macrophage accumulation, we analyzed expression of multiple cytokines during cyst progression with quantitative real-time PCR (qRT-PCR) from whole kidney lysates. Cytokines including CCL2, CSF1 and CX3CL1 are associated with inflammation in cystic kidney diseases (Zimmerman et al., 2019b; Cassini et al., 2018; Zimmerman et al., 2020; Ta et al., 2013). In agreement with minimal macrophage accumulation at 6 wpi, there was no difference in expression of these cytokines between controls and *Pkd2* mutants. However, *Ccl2* and *Csf1* expression increased in *Pkd2* mutant kidneys at 12 wpi and increased further at 16 wpi, which aligned with the changes in macrophage populations. *Cx3cl1* expression was significantly increased in *Pkd2* mutants compared to controls only at 16 wpi (Fig. 5E). This suggests that increased cytokine signaling coincides with the initiation and formation of cysts.

### Preventing macrophage accumulation ameliorates cyst progression in non-injured adult-induced *Pkd2* mutant kidneys

**Fig. 6.**
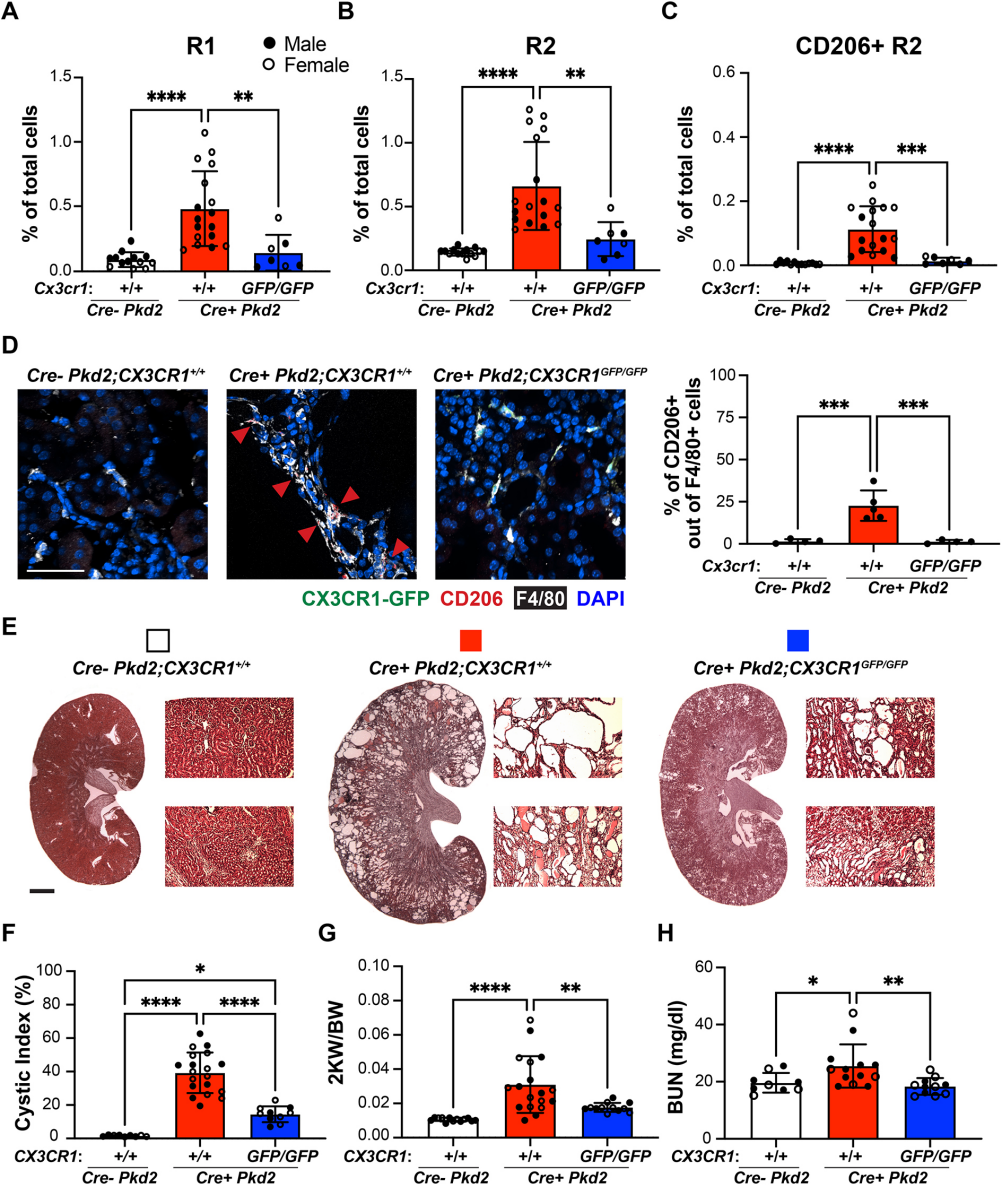
**Preventing macrophage accumulation using *Cx3cr1* disruption significantly suppresses cyst progression in non-injured adult-induced *Pkd2* mutant kidneys.** (A-C) Bar graphs showing the percentages of infiltrating R1 macrophages (A), resident R2 macrophages (B) and CD206^+^ R2 macrophages (C) of total renal cells in *Cre^−^ Pkd2;Cx3cr1^+/+^* (*Cre^−^ Pkd2*), *Cre^+^ Pkd2;Cx3cr1^+/+^* (*Cre^+^ Pkd2*, +/+) and *Cre^+^ Pkd2;Cx3cr1^GFP/GFP^* (*Cre^+^ Pkd2*, GFP/GFP) mice that were induced at the age of 8 weeks and analyzed at 16 wpi by flow cytometry analysis. (D) (Left) Representative confocal images showing CX3CR1-GFP^+^ cells (green), and CD206 (red), F4/80 (white) and DAPI (blue) staining for each group induced at 16 wpi. Red arrowheads indicate locations of CD206^+^ macrophages. Scale bar: 50 μm. (Right) Bar graph showing the percentage of CD206^+^ of F4/80^+^ cells from immunofluorescent staining. (E) Representative H&E-stained kidney images, and 10× magnification images for cortex (top row) and medulla (bottom row) from each group. Scale bar: 1 mm. (F-H) Bar graphs showing the cystic index (F), 2KW/BW (G) and blood urea nitrogen (BUN) (H) in each group at 16 wpi. Each dot represents an individual mouse; filled circles and open circles represent male and female mice, respectively. Error bars represent ±s.d. *n*≥10 for each group. *P<0.05, **P<0.01, ***P<0.001 and ****P<0.0001 by one-way ANOVA.

The data above raised the possibility that CD206^+^ resident macrophages are associated with phases of rapid cyst growth. CX3CR1 is a chemokine receptor, the expression of which is enriched in resident macrophages, including those expressing CD206 (Liu et al., 2020; Lionakis et al., 2013; Ide et al., 2020; Yona et al., 2013; Stremmel et al., 2018). The CX3CR1/CX3CL1 signaling axis controls the maintenance and accumulation of resident macrophages (Liu et al., 2020). Our previous data indicated that CD206 is expressed to a very limited extent by infiltrating macrophages under normal quiescent conditions (Zimmerman et al., 2019a). Thus, we crossed *Cx3cr1* null mice (*Cx3cr1^GFP/GFP^*) to the *Pkd2* conditional mutants (Cre^+^ *Pkd2*) to prevent the accumulation of resident macrophages, including the CD206^+^ subtype, and evaluated the impact on cyst formation in the adult-induced mutant kidneys. Importantly, this was done in the absence of an exogenous injury. Flow cytometry analysis showed a substantial decrease in the number of resident macrophages, but unexpectedly also in the infiltrating population, in adult-induced *Cre^+^ Pkd2;Cx3cr1^GFP/GFP^* compared to *Cre^+^ Pkd2;Cx3cr1^+/+^* at 16 wpi (Fig. 6A,B). Notably, the loss of *Cx3cr1* prevented the re-accumulation of the CD206^+^ resident macrophages as determined by flow cytometry and IF microscopy analysis (Fig. 6C,D; Fig. S6B). The number of CD206^+^ infiltrating macrophages did not change in *Pkd2* mutant kidney with or without *Cx3cr1*, although the number of CD206^+^ infiltrating macrophages was extremely low compared to CD206^+^ resident macrophages (Fig. S6A). More importantly, analysis of renal histology, cystic index, 2KW/BW and blood urea nitrogen (BUN) indicated that the cyst progression was attenuated, and renal function was improved, in *Pkd2* mutant kidneys in the absence of *Cx3cr1* (Fig. 6E-H). In addition, although body weight was reduced in the *Pkd2* mutants compared to normal controls, it was similar between *Pkd2* mutants with or without *Cx3cr1* (Fig. S6C). Collectively, these data suggest that CX3CR1^+^ macrophages, and a CD206^+^ subset of these macrophages, are closely associated with cyst formation in the adult-induced *Pkd2* mutants. Importantly, the effect of reducing cyst formation by preventing macrophage accumulation occurs in the absence of a renal injury. In previous studies, reducing the number of macrophages following the injury may have simply reduced the level of injury the kidneys were experiencing (Zimmerman et al., 2019b, 2020).

### A subset of CD206^+^ resident macrophage-like cells is conserved in mouse and human kidneys

**Fig. 7.**
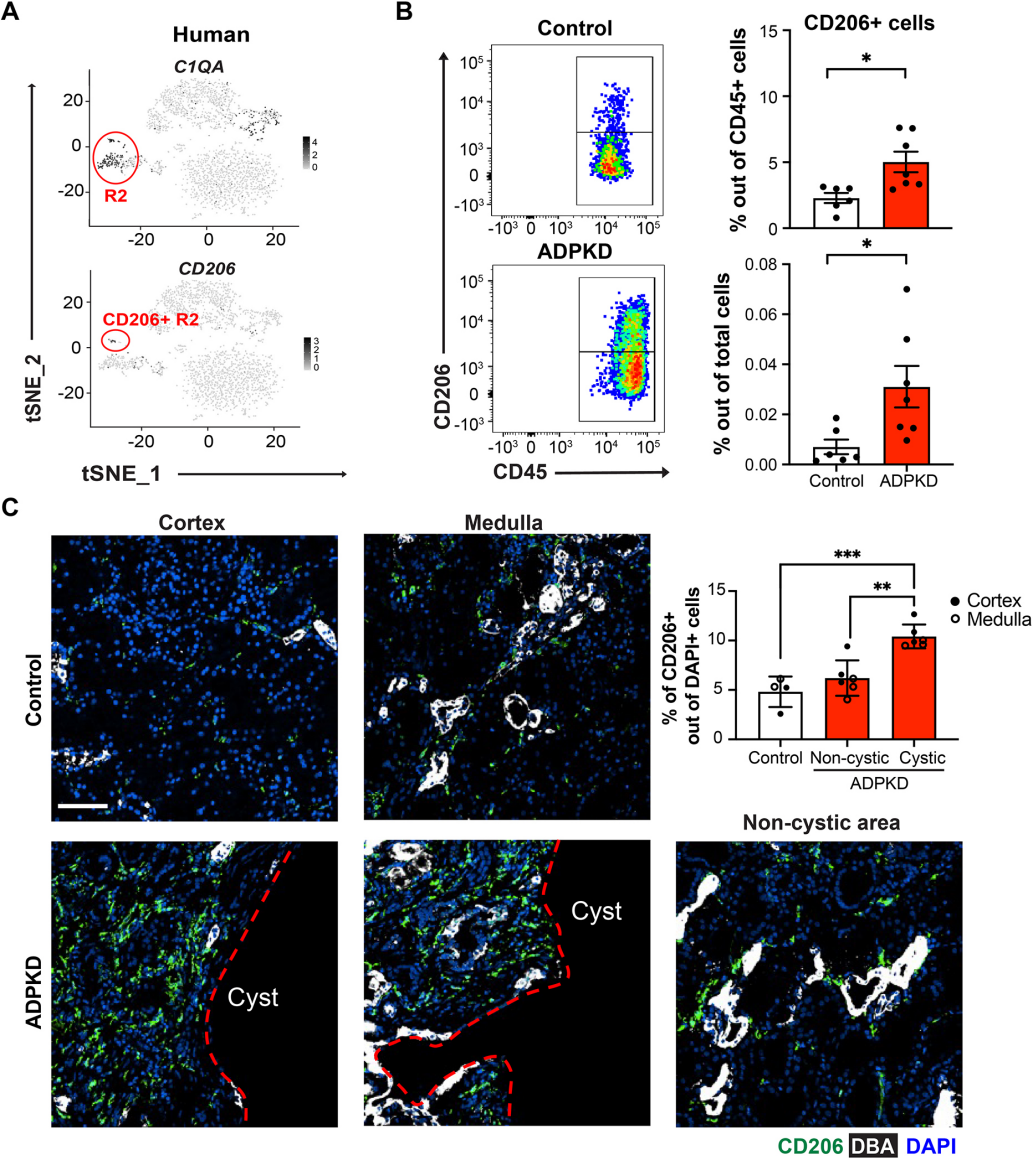
**The number of CD206^+^ resident macrophages is increased in autosomal-dominant polycystic kidney disease (ADPKD) patient kidneys.** (A) t-distributed stochastic neighbor embedding (t-SNE) plots of scRNA-seq data of innate immune cells from normal human kidneys. t-SNE depicting genes used to identify resident macrophages (*C1QA*) and CD206^+^ resident subpopulation of macrophages (*CD206*). (B) (Left) Representative flow cytometry plots and quantification of CD206^+^ cells isolated from control (*n*=6) and ADPKD kidneys (*n*=7). (Right) Quantification of each population from control (white bars) and ADPKD kidneys (red bars) is shown as a percentage of CD45^+^ immune cells (top) and a percentage of total renal cells (bottom). Each dot represents individual patients. *P<0.05 by unpaired two-tailed Student's *t*-test. (C) Representative immunofluorescence images demonstrating increased CD206^+^ macrophage accumulations in cystic regions of cortex and medulla from ADPKD patients compared with non-cystic areas. Red dashed lines demarcate the cystic and non-cystic areas. In contrast, minimal CD206^+^ macrophage accumulation was observed in control kidney. CD206 (green), DBA (white) and DAPI (blue) staining is shown. Scale bar: 100 μm. (Top right) Bar graph showing the percentage of CD206^+^ macrophages of the total nucleus number. Error bars represent ±s.d. *n*≥4 for each group. **P<0.01 and ***P<0.001 by one-way ANOVA.

Our recent studies and data shown here indicate that CD206 is a marker of resident macrophages in mouse kidneys (Zimmerman et al., 2019a) (Fig. 1B). To determine whether this was also true in normal human kidney tissue, we re-analyzed our previously published scRNA-seq dataset [Gene Expression Omnibus (GEO): GSE128992] that contained innate immune cells from normal adult human kidneys (Zimmerman et al., 2019a). As expected, resident macrophages were also detected in human kidneys, as indicated by enriched *C1QA* expression, and within this population we detected a small subset that expressed CD206 (Fig. 7A). The small number of CD206^+^ cells was expected based on the rare presentation of CD206^+^ R2 in adult mouse kidney. These data suggest that a conserved population of resident macrophages with CD206 expression exists in human kidneys (referred to as CD206^+^ resident macrophage-like cells).

### CD206^+^ resident macrophage-like cell numbers are increased in human ADPKD kidneys

Because we observed an association between the number of CD206^+^ resident macrophages and rapid cyst progression in mice, we investigated possible parallels in human ADPKD. We performed flow cytometry analysis of ADPKD kidneys and non-ADPKD controls (Fig. S7; Table S1). As in mice, the data indicate that the number of CD206^+^ resident macrophage-like cells is significantly increased in ADPKD kidneys compared to controls (Fig. 7B). Surprisingly, our flow cytometry data showed a nearly equal distribution of CD206^+^ resident macrophage-like cells in renal tissues from the cortex and medulla in human ADPKD kidneys (Fig. S8). Analysis of IF confocal images revealed a substantial accumulation of CD206^+^ cells adjacent to renal cysts but not around non-cystic areas (Fig. 7C). As shown in our flow cytometry and scRNA-seq data, we observed very few CD206^+^ cells in tissue sections of the non-ADPKD control kidneys.

### CD206^+^ resident macrophage-like cells are present in the urine of ADPKD patients, and their numbers correlate with the rate of renal function decline

**Fig. 8.**
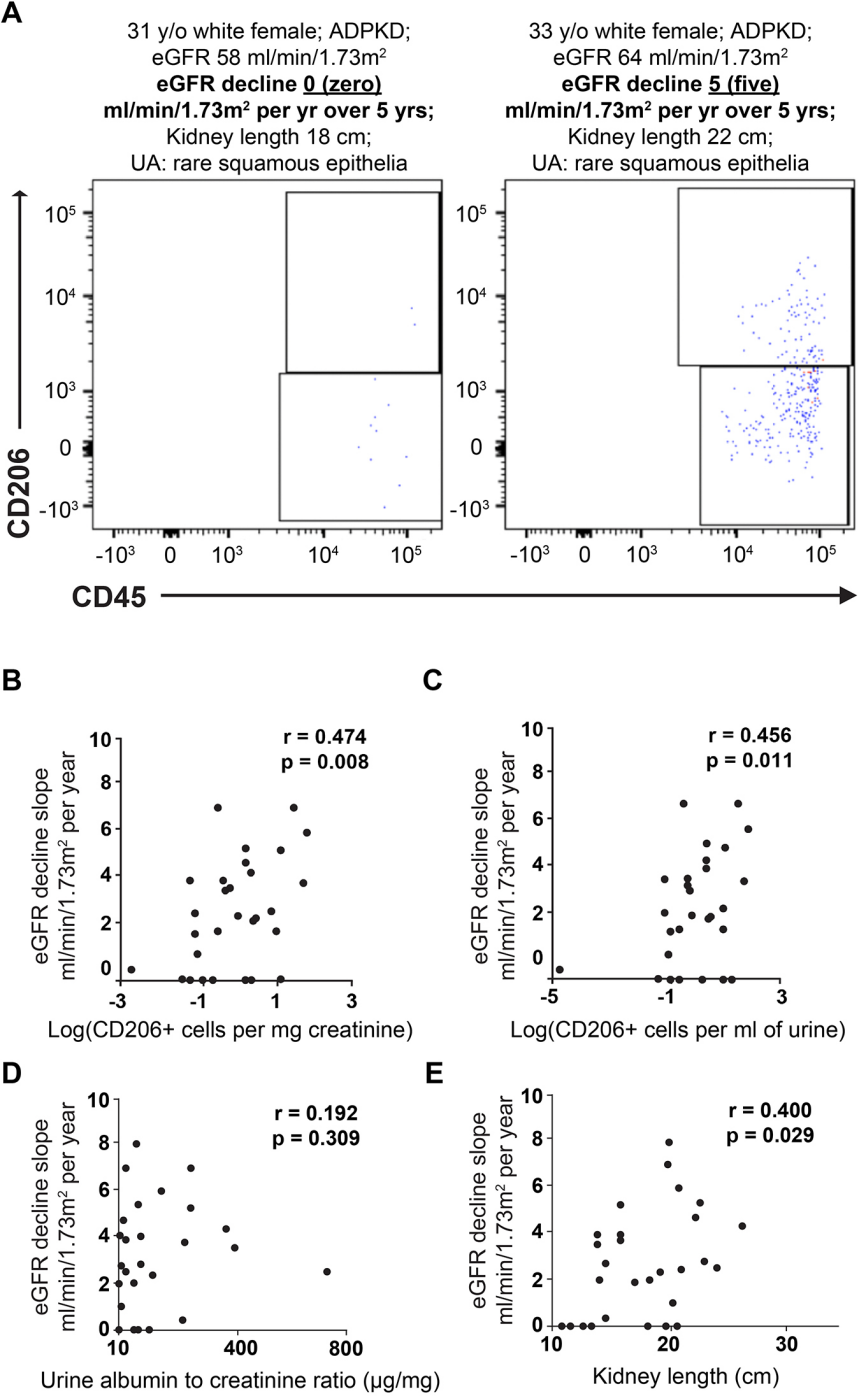
**Urinary CD206^+^ macrophages correlate with glomerular filtration rate (eGFR) decline in ADPKD kidneys.** (A) Representative flow cytometry plots of urinary CD206^+^ macrophages from ADPKD patients with similar demographic characteristics and kidney length but a different rate of eGFR loss over time. (B-E) Scatterplots representing the correlation between the average annual eGFR loss and urinary CD206^+^ macrophage-like cell indices normalized by urine creatinine level (B), urinary CD206^+^ macrophage-like cell indices normalized by urine volume (C), kidney length (D), and urine albumin to creatinine ratio (E). Each dot represents an individual patient. *n*=30. UA, urinalysis.

Although we observed a higher number of CD206^+^ resident macrophage-like cells in kidneys from end-stage ADPKD patient kidneys, we could not determine whether these cells were also more abundant in ADPKD patients with earlier pre-end-stage kidney disease (ESKD), given the limited availability of such tissues. Because resident macrophages can exert a dynamic migratory behavior (Kierdorf et al., 2015; Neupane et al., 2020), we speculated that renal CD206^+^ resident macrophage cells might migrate into renal tubules and be carried away with the urine flow. Thus, we tested whether the number of CD206^+^ cells in urine reflects the magnitude of renal cystogenic activity. Flow cytometry analysis confirmed this concept in a small cohort of consecutively collected urine samples from patients with ADPKD (*n*=30). CD206^+^ resident macrophage-like cells were detected in the urine of all 30 ADPKD patients. Notably, a higher number of CD206^+^ resident macrophage-like cells was present in urine from patients with a more progressive rate of kidney function loss (Fig. 8A). The analysis of urine cells from pre-ESKD ADPKD patients revealed a moderately strong correlation between the urine CD206^+^ resident macrophage-like cell index and the average annual glomerular filtration rate (eGFR) decline over 5 years (*r*=0.474, *P*=0.008; Fig. 8B). Adjustment for urine volume instead of creatinine concentration yielded a similar result (*r*=0.456, *P*=0.011; Fig. 8C). In comparison, urine albumin to creatinine ratio, a widely used biomarker and an established predictor of ADPKD progression, correlated weakly with the eGFR decline slope (*r*=0.192, *P*=0.309; Fig. 8D). Together, these results suggest that urinary CD206^+^ resident macrophage-like cell number is a candidate biomarker of the ADPKD renal disease activity and predictor of the rate of future eGFR decline.

As previously reported, the correlation between kidney length [KL; a recently identified predictor of ADPKD progression (Bhutani et al., 2015)] and the slope of eGFR decline in this patient cohort showed a moderately strong association (*r*=0.400, *P*=0.029; Fig. 8E). Because the structural renal cystic burden indices (e.g. KL) reflect a cumulative effect of the disease activity that is exerted over years, the weaker and insignificant association between the cumulative cystic burden index and CD206^+^ indices further support the potential utility of the urinary CD206^+^ resident macrophage-like cells as a marker of the disease activity in ADPKD.

## DISCUSSION

The rate of cystogenesis is influenced by the time at which the polycystin proteins are inactivated in PKD mouse models, with cyst growth rates being much faster when polycystins are inactivated in juvenile kidneys compared to adult-induced mutant kidneys. This suggests that a critical switch occurs during renal maturation and that it defines the kinetics of renal cyst formation. Given that renal macrophages also undergo a rapid phenotypic switch during kidney maturation, we tested whether a subset of renal macrophages contributes to the different rates of cyst formation in juvenile and adult mutant kidneys. We identified a subset of resident macrophages, but not infiltrating macrophages, with CD206 expression (referred to as CD206^+^ R2) that is present during rapid cyst formation. In juvenile kidneys, CD206^+^ R2 cells are already present, and induction of the *Pkd2* mutation leads to rapid cyst progression. IF microscopy analysis showed that CD206 macrophages accumulate in areas of cyst development. In contrast, normal adult kidneys have very few CD206^+^ R2 macrophages, and loss of *Pkd2* in adult-induced mice results in cyst formation in a slow and focal manner, and the CD206^+^ R2 macrophages only accumulate in areas of focal cyst formation.

The CX3CR1/CX3CL1 signaling axis is essential for macrophage survival and accumulation, and loss of *Cx3cr1* predominately affects resident macrophage regeneration under normal and pathological conditions (Lionakis et al., 2013; Ide et al., 2020). Thus, we attempted to prevent resident macrophage accumulation, including those expressing CD206, using the *Cx3cr1* null mice. With this model, we observed that mutation of *Cx3cr1* resulted in significant reduction in the number of CD206^+^ R2 and led to markedly attenuated cyst severity in *Pkd2* mutant kidney in the absence of *Cx3cr1*. We hypothesize that CD206^+^ resident macrophages are important contributors to rapid cyst formation; however, we are not able to confirm a direct function of CD206^+^ resident macrophages because the deletion of *Cx3cr1* significantly reduced the numbers of both resident and infiltrating macrophage populations in the adult-induced *Pkd2* mutant kidneys. A possible explanation for the reduced number of infiltrating macrophages in the *Cx3cr1* mutants could be that resident macrophages have a role in recruiting infiltrating macrophage from bone marrow to kidney. Further investigation to specifically deplete CD206 resident macrophages will be needed to test the direct role of this population in cyst formation.

We previously demonstrated that ischemia–reperfusion injury in adult-induced cilia mutant kidneys greatly accelerates cystogenesis and that this is associated with re-accumulation of juvenile-like resident macrophages. Furthermore, inhibition of resident macrophage proliferation using a CSF1 receptor antagonist reduced cyst severity in adult-induced *Ift88* mutants following injury (Zimmerman et al., 2019b). Because macrophages play a major role in mediating the injury response in the kidney, it was uncertain whether the reduction in cyst severity was simply a result of a reduced level of the injury associated with the presence of fewer macrophages or a more direct effect of macrophages on cyst expansion. Importantly, here we show a similar impact on cyst growth related to the reduction of CX3CR1^+^ macrophages in the adult-induced models in the absence of any exogenous injury. These data support a more direct role of macrophages in cyst formation in adult-induced mutants.

Using scRNA-seq, we previously showed that a population of innate immune cells in rats, pigs and humans shares a core gene expression signature with mouse resident macrophages (Zimmerman et al., 2019a). *C1QA* is one of the genes identified that is highly enriched in resident macrophage-like cells in human kidney. These data are supported by a recent study from Stewart et al. (2019), which showed enriched *C1QC* expression in a small cluster of mononuclear phagocytes from the human kidney, and we previously demonstrated in rat parabiosis studies that these are likely resident macrophage and not bone marrow-derived infiltrating macrophages (Zimmerman et al., 2021). These findings suggest that resident macrophages are present in the kidney across species, including in humans. Our scRNA-seq analyses identified a small subset of *C1QA*^+^ resident macrophages in normal human kidneys that also expresses *CD206*. This suggests that CD206^+^ resident macrophage-like cells in mice and humans may be similar. More importantly, our analysis of human ADPKD patient kidneys and normal kidneys demonstrated a correlation between CD206^+^ macrophages and cystic severity as in the mouse models. As in mice, the CD206^+^ cells were barely detectable in controls or non-cystic samples and accumulated in areas around developing cysts. Importantly, the CD206^+^ resident macrophage-like cells were also present in urine samples from pre-ESKD ADPKD patients, and indices reflecting their numbers correlated with the slope of eGFR decline, an indicator of the disease activity. The indices of urinary CD206^+^ resident macrophage-like cells represent an attractive non-invasive candidate biomarker of renal ADPKD activity. These indices might also well complement the kidney size-based indices in predicting near-future ADPKD outcomes.

## MATERIALS AND METHODS

### Mice

*CAG-Cre^ERT2^ PKD2^fl/fl^ mice* on a C57BL/6J background were bred in-house. *Cx3cr1^GFP/GFP^* mice (Stock No: 005582) and *ROSA^mTmG^* mice (Stock No: 007576) were purchased from The Jackson Laboratory. For adult induction of *Pkd2* deletion, 8-week-old male and female mice were given intraperitoneal injections of tamoxifen at 6 mg/40 g body weight once daily for 3 consecutive days. For *Pkd2* deletion in juvenile mice, nursing mothers were given one intraperitoneal injection of tamoxifen at 9 mg/40 g body weight at the indicated time point. Deletion of *Pkd2* was confirmed when animals were harvested at the indicated time points by PCR and qRT-PCR. The following primers were used to detect *Pkd2* by PCR: 5′-GCTGCTGCCCTTTCCTCTGTG-3′ (5′ primer), 5′-CTGACAGGCACCTACAGAACAGTGA-3′ (3′ primer), 5′-TGAAAGTTTGATGCTTAGCAGATGATGGC-3′ (delta primer). A TaqMan probe (Thermo Fisher Scientific) was used to detect *Pkd2* in qRT-PCR (Assay ID: Mm00435841_m1).

Animals were maintained in Associated for Assessment and Accreditation of Laboratory Animal Care International-accredited facilities in accordance with Institutional Animal Care and Use Committee regulations at the University of Alabama at Birmingham (UAB) (approval number: 21072).

### Hematoxylin and Eosin (H&E) staining and cystic index calculation

Mouse kidneys were harvested and fixed in 4% paraformaldehyde (PFA) overnight at 4°C, then immersed in 70% ethanol overnight. Kidney tissues were embedded in paraffin, sectioned at 10 μm and stained using H&E as previously described (Zimmerman et al., 2019b). The whole-kidney images were obtained with a Nikon Eclipse TE2000 Microscope, and the cystic indices of whole-kidney sections were calculated. Briefly, cystic area and whole-kidney area were measured using ImageJ. The cystic index was defined as the area percentage of cysts over the total kidney area. The final cystic index of each mouse was determined by the mean of results of three to four sections from each kidney.

### IF staining and microscopy

Following fixation in 4% PFA overnight, half of the right kidney was cryoprotected in 30% sucrose overnight, embedded in Optimal Cutting Temperature (OCT) medium and sectioned at 10 μm. For IF staining, sections were fixed with 4% PFA for 10 min, permeabilized with 0.2% Triton X-100 for 10 min and incubated in blocking solution [1% bovine serum albumin (BSA), 2% donkey serum and 0.02% sodium azide into 1× PBS] for 30 min at room temperature. Sections were incubated in primary antibody overnight at 4°C according to the manufacturer's recommendations, washed with PBS and incubated with the appropriate secondary antibodies in blocking solution for 1 h at room temperature (primary and secondary antibodies are listed in Table S2). After the addition of secondary antibodies, nuclei were stained by Hoechst (Sigma-Aldrich), and samples were mounted using IMMU-MOUNT (Thermo Fisher Scientific). All fluorescence images were captured on a Nikon Spinning-disk confocal microscope with a Yokogawa X1 disk, using a Hamamatsu flash4 sCMOS camera. Images were processed and analyzed using NIS Elements software (Nikon) version 5.0 and ImageJ.

### Flow cytometry analysis of macrophages from mouse kidney tissues

Samples were processed as described in detail previously (Zimmerman et al., 2019b). Briefly, the left kidney was harvested after perfusion of the mouse with 1× PBS. Kidney tissues were minced and digested in 1 ml RPMI 1640 (Gibco, Grand Island, NY, USA) containing 1 mg/ml collagenase type I (Sigma-Aldrich, C0130) and 100 U/ml DNase I (Sigma-Aldrich, D5025) for 30 min at 37°C with agitation. Kidney fragments were then passed through a 70 μm mesh (Falcon, 352350) to dissociate single-cell suspensions. Red blood cells were lysed with ACK lysing buffer (Quality Biological, 118-156-101) and subsequent centrifugation at 500 ***g*** for 5 min, and cells were resuspended in 1 ml 1× PBS containing 1% BSA with Fc blocking solution (BioXcell, BE0307) with 1:200 dilution in PBS for 30 min on ice. Approximately 2 million cells were stained for 30 min at room temperature with the fluorescent dye-conjugated antibodies listed in Table S2. Cells were washed with 1× PBS, fixed in 2% PFA for 30 min and resuspended in FACS (1% BSA in 1× PBS) buffer. After immunostaining, cells were analyzed on a BD LSRII Flow Cytometer (BD Biosciences). Data analysis was performed using FlowJo version 10.8.1 software.

### qRT-PCR

RNA was isolated using Qiagen RNeasy Plus kit (or TRIzol) and subjected to cDNA preparation with High-Capacity cDNA Reverse Transcription Kit (Thermo Fisher Scientific, 4368814). qRT-PCR was performed using a CFX Connect Real-Time System (Bio-Rad) with TaqMan™ Gene Expression Master Mix (Thermo Fisher Scientific, 4369016) according to the manufacturers’ instructions. TaqMan probes were used for qRT-PCR, including *Hprt* (Assay ID: Mm00446968_m1), *Ccl2* (Assay ID: Mm00441242_m1), *Cx3cl1* (Assay ID: Mm00436454_m1) and *Csf1* (Assay ID: Mm00432686_m1). Gene expression analysis was performed using the comparative 2^−(ΔΔCt)^ method with *Hprt* for normalization.

### Human kidney tissue sample collection

This study was performed according to the principles expressed in the Declaration of Helsinki. Remnant human ADPKD and non-PKD kidney tissues and urine samples were collected, de-identified and analyzed according to a protocol (IRB-131223003) approved by the Institutional Review Board of the UAB. ADPKD tissues were obtained from patients with ESKD (all pre-transplant). All non-PKD kidney tissues were collected from patients with intra-renal tumors, as previously described (Zimmerman et al., 2019c). The eGFR estimated by Chronic Kidney Disease Epidemiology Collaboration (CKD-EPI) formula (Shen et al., 2017) in non-PKD controls was in the range of 58-120 (average 89) ml/min/1.73m^2^ (Table S1). The average age of ADPKD patients was lower (53±11 years) than that of non-PKD controls (58±13 years; *P*=0.542). Tissues from the cortex and medulla of PKD and non-PKD tissue were harvested and validated as specific to the cortex and medulla as previously described (Zimmerman et al., 2019c). Tissues from immediate sub-capsular and corticomedullary regions, as well as renal papilla, were excluded.

### Human urine sample collection

Remnant urine samples were collected from consecutive patients with ADPKD diagnosis established by family history and imaging criteria; patients with suspected systemic, renal or urinary tract infection based on history, exam, laboratory or imaging studies (including urinalysis) were excluded. These urine samples were procured and analyzed within 4 h of collection using the same flow cytometry technique for kidney tissues as previously described (Zimmerman et al., 2019c). Relevant demographic, laboratory and imaging data were recorded for 30 patients that formed the studied cohort (age 49±13.5 years; male, 30%; African American, 17%; Caucasian, 83%; eGFR decline 3±2.4 ml/min/1.73 m^2^/year). The results that were not obtained at the urine collection (e.g. kidney size) were based on time-adjusted earlier measurements.

### Flow cytometry of macrophages from human kidney tissues

Flow cytometry was performed on ∼2-3 g of kidney tissue from either the renal cortex or medulla as described in our previous study (Zimmerman et al., 2019c). The single-cell suspension was processed similar to the mouse kidney tissues described above. Cells were incubated in 1% BSA containing the following antibodies (Table S2): eFluor450 mouse anti-human CD45, BV650 mouse anti-human HLA-DR1, BV786 mouse anti-human CD206 and aqua fluorescent reactive dye (Invitrogen, L34957). Cells were washed with 1× PBS and re-suspended in FACS buffer. All samples were analyzed on the LSRII flow cytometer (BD Biosciences) and FlowJo version 10.0 software.

### Flow cytometry of macrophages from human urine

Flow cytometry on urinary cells was done using 20-50 ml remnant urine samples within 4 h of collection. The processing and isolation of urinary cells was done according to the previously described protocol (Zimmerman et al., 2019c). Briefly, cells were isolated by centrifugation at 500 ***g*** for 10 min, washed in 1% BSA and re-spun at 500 ***g*** for 5 min at 4°C. The isolated cells were stained for 30 min at room temperature with the antibodies (as listed above for flow cytometry analysis for human kidney tissues). Next, cells were centrifuged at 500 ***g*** for 5 min at 4°C, washed with BSA and fixed in 2% PFA for 30 min at room temperature. The labeled cells were re-suspended in FACS buffer and analyzed using the LSRII flow cytometer.

### Statistical analysis

Statistical evaluations, including outcome distributions and correlations for human data, were performed with SPSS 23.0 software package. GraphPad Prism 9 was used for the generation of all graphs and statistical tests. All results were presented as mean±s.d. Numbers and statistical tests are indicated in figure legends. All experiments involving phenotyping of mutants were scored blind to genotype, then corresponded with the genotype.

## Supplementary Material

10.1242/dmm.049810_sup1 Supplementary information
