# Supplementary material for: A kidney resident macrophage subset is a candidate biomarker for renal cystic disease in preclinical models
Source: Dis Model Mech. 2023 Jan 16;16(1):dmm049810. doi: 10.1242/dmm.049810 (PMC9884121; doi:10.1242/dmm.049810)
Supplement: Supplementary information [file dmm-16-049810-s1.pdf]

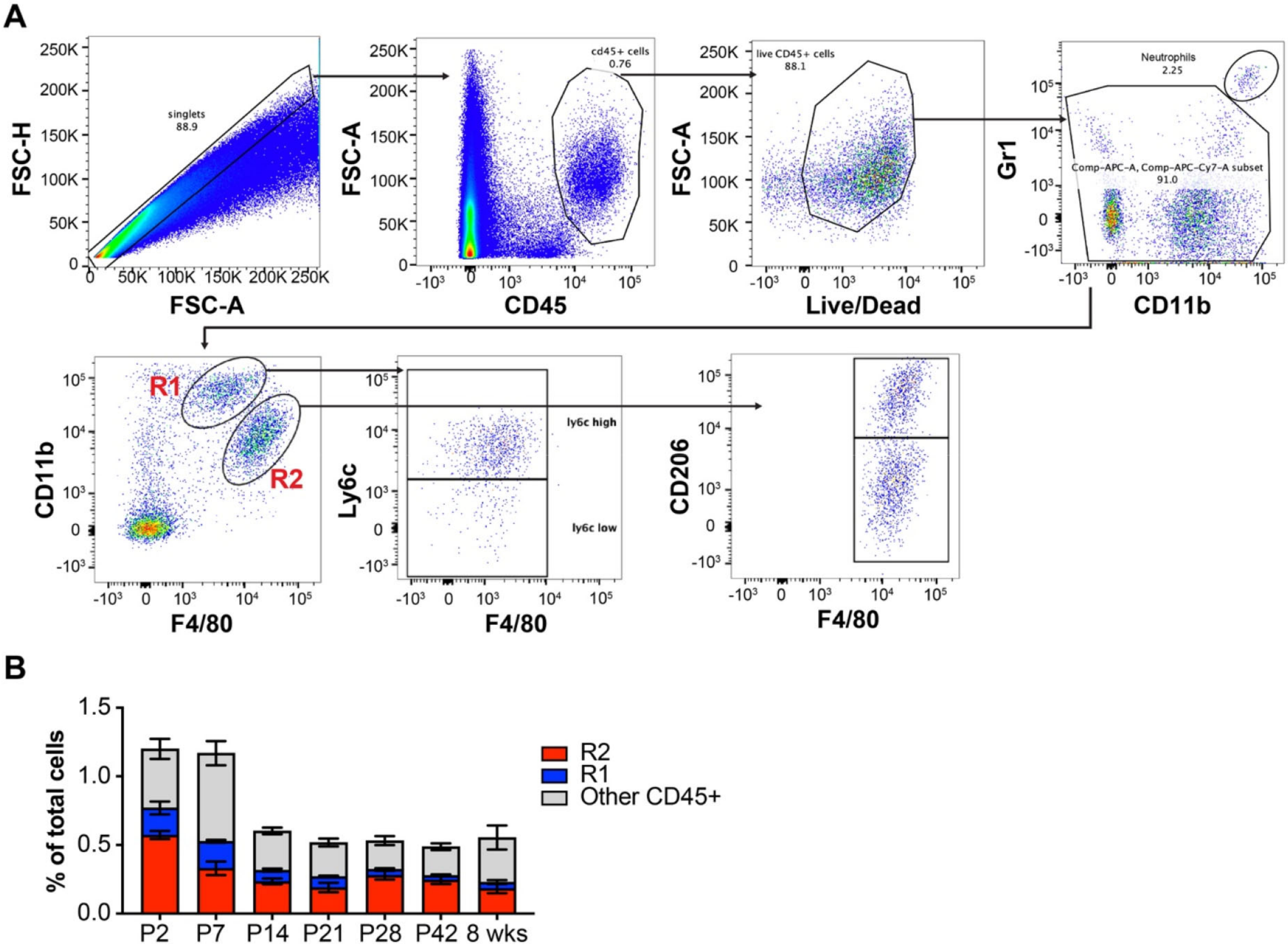

Fig. S1. Infiltering and resident macrophage population during postnatal kidney maturation. (A) Flow cytometry gating strategy for infiltrating macrophages (R1) and its sub-populations expressing Ly6c and resident macrophage (R2) and its sub-populations expressing CD206. (B) Stacked bar graph showing the percentage of R1 and R2 in total renal cells from wild-type (WT) kidney at P2, P7, P14, P21, P28, P42 and 8 wks (adult) with flow cytometry analysis.

Disease Models & Mechanisms • Supplementary information

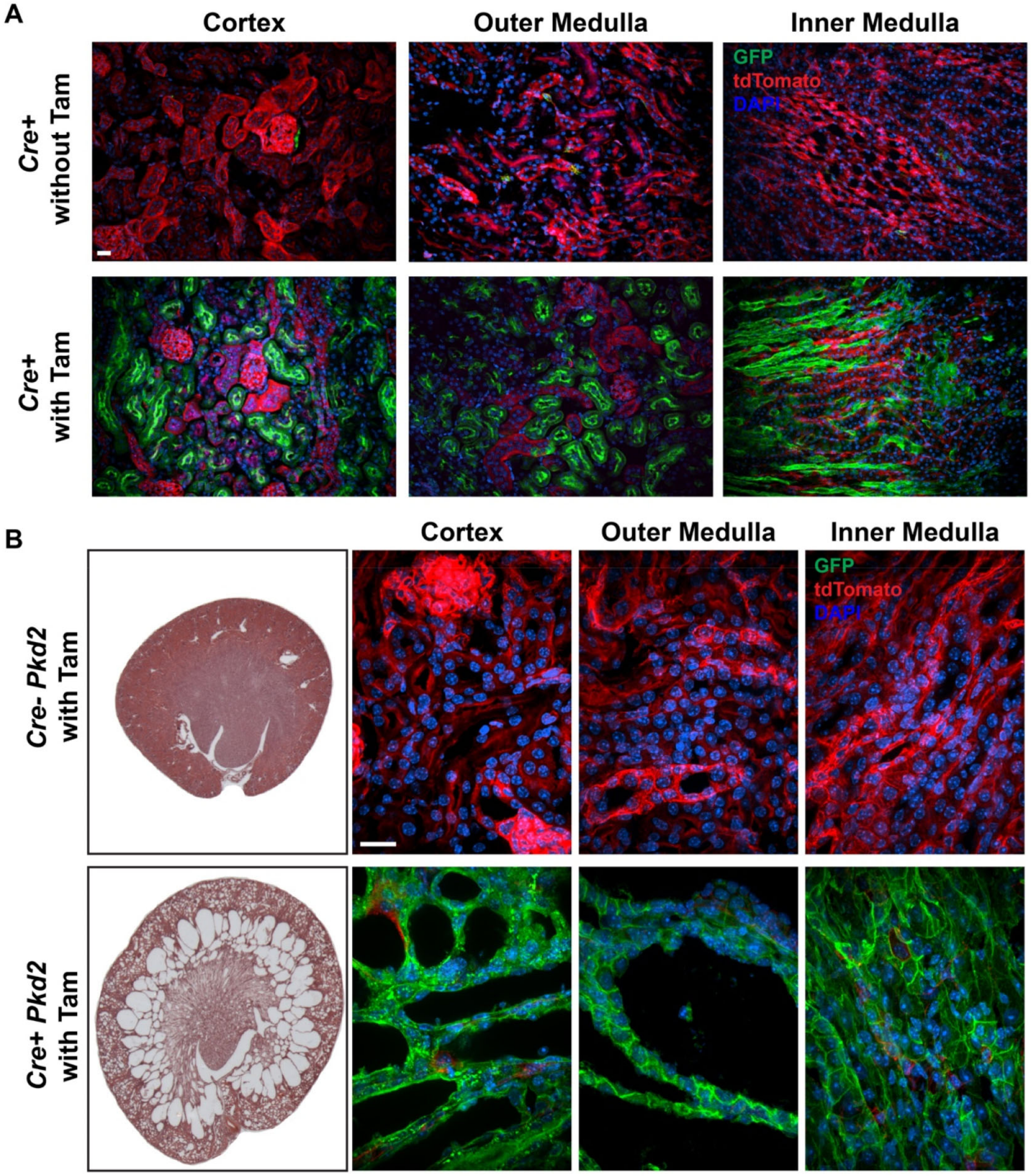

Fig. S2. A high efficiency of *Cre* recombination with Tamoxifen induction. (A) Representative confocal images showing GFP (green), tdTomato (red) and nucleus (DAPI, blue) in the OCT-embedded cortex and medulla regions of the kidney from *Cre+ mTmG* mice with and without tamoxifen at P7 induction post 2 weeks. (B) Representative H&E-stained kidney sections images and confocal images showing histological changes, GFP and tdTomato expression from *Cre- Pkd2/mTmG* and *Cre+ Pkd2/mTmG* mice at P7 induction post 2 weeks. Scale bar: 20μm.

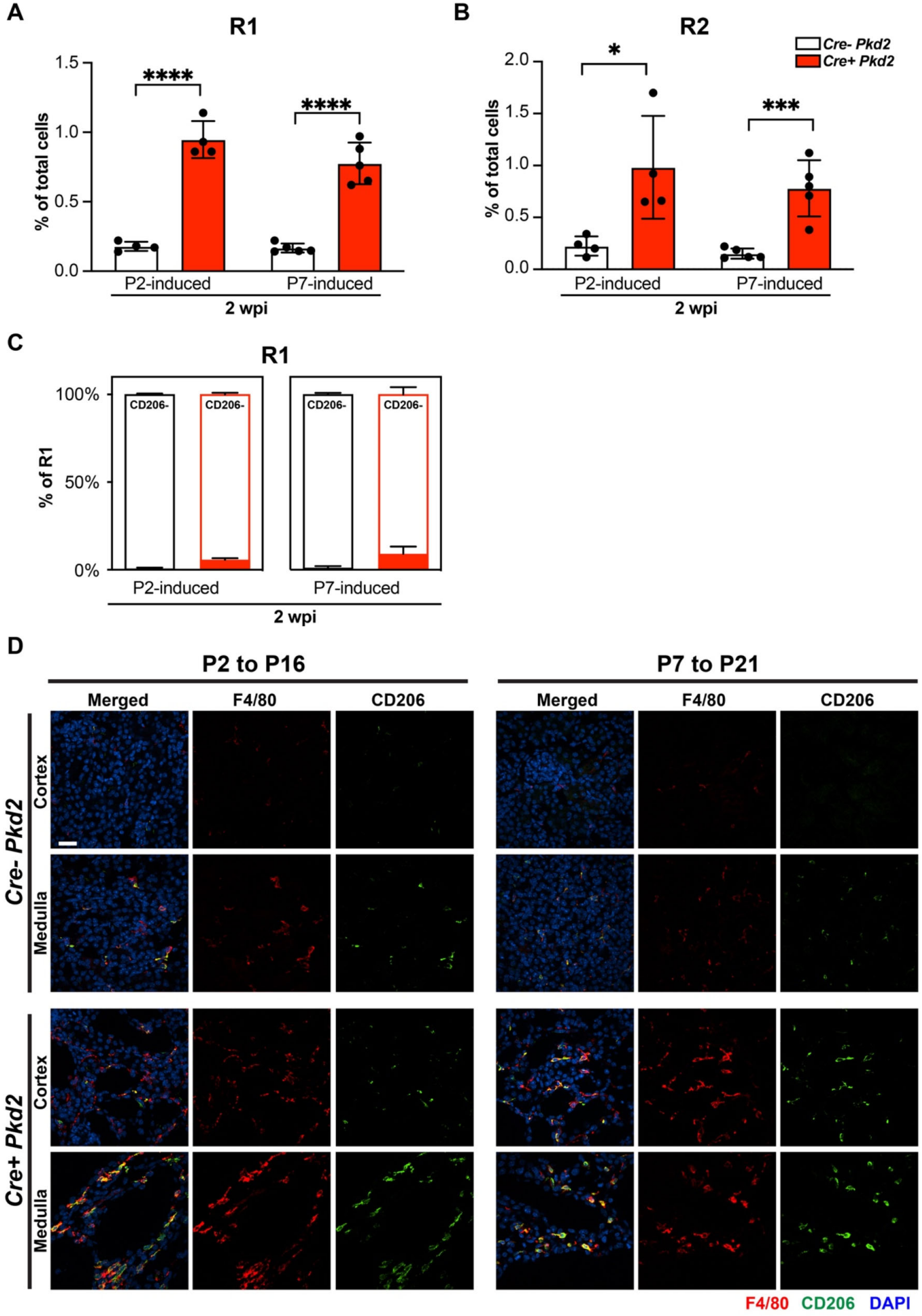
A
R1
% of total cells
1.5
1.0
0.5
0.0
****
****
P2-induced
P7-induced
2 wpi
B
R2
Cre- Pkd2
Cre+ Pkd2
2.0
1.5
1.0
0.5
0.0
*
***
P2-induced
P7-induced
2 wpi
C
R1
100%
50%
0%
% of R1
CD206-
P2-induced
P7-induced
2 wpi
D
P2 to P16
P7 to P21
Merged
F4/80
CD206
Cre- Pkd2
Cre+ Pkd2
Cortex
Medulla
F4/80 CD206 DAPI

Fig. S3. Increased numbers of infiltrating and resident macrophages in juvenile induced *Pkd2* mutant kidneys with rapid cyst formation. The flow cytometry analysis of the percentage of R1 macrophages (A) and R2 macrophages (B) out of total renal cells from P2 and P7 induced kidney analyzed at 2 weeks post induction (wpi). Each dot represents an individual mouse. Error bars represent ±SD. P<0.05 *, P<0.001 ***, P<0.0001 **** as determined using an unpaired t-test. (C) The percentage of CD206+ and CD206- R1 in total infiltrating macrophages from P2 and P7 induced kidney analyzed at 2 wpi. Error bars represent ±SD. (D) Representative merged and split images showing F4/80 (red), CD206 (green) and DAPI (blue) staining in cortex and medulla for *Cre- Pkd2* and *Cre+ Pkd2* mice induced at P2 or P7 and analyzed at 2 wpi. Scale bar: 20μm.

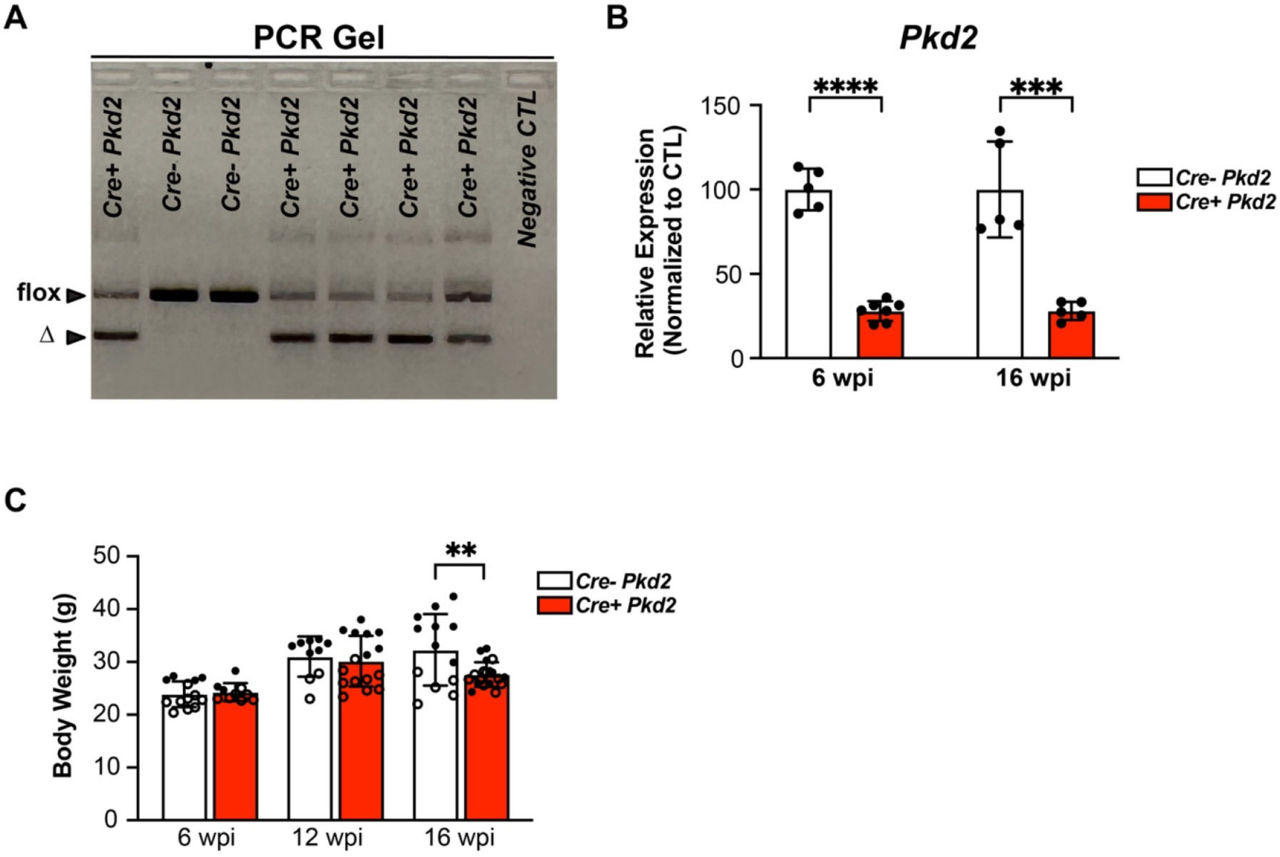

Fig. S4. Tamoxifen induction in adult *Pkd2* mutant mice. (A) Gel image showing the efficiency of *Pkd2* deletion after Tamoxifen at 16 wpi. The floxed *Pkd2* allele (300bp) and Δ allele (200bp) pointed out. (B) The relative mRNA expression of *Pkd2* from whole-kidney lysates of *Cre- Pkd2* and *Cre+ Pkd2* at 16 wpi with qRT-PCR analysis. (C) Bar graph showing body weight change in *Cre- Pkd2* and *Cre+ Pkd2* at indicated time points. Each dot represents an individual mouse with solid circles and empty circles representing male and female mice, respectively. Error bars represent ±SD. P<0.01 **, P<0.001 *** and P<0.0001 **** by unpaired t-test.

Disease Models & Mechanisms • Supplementary information

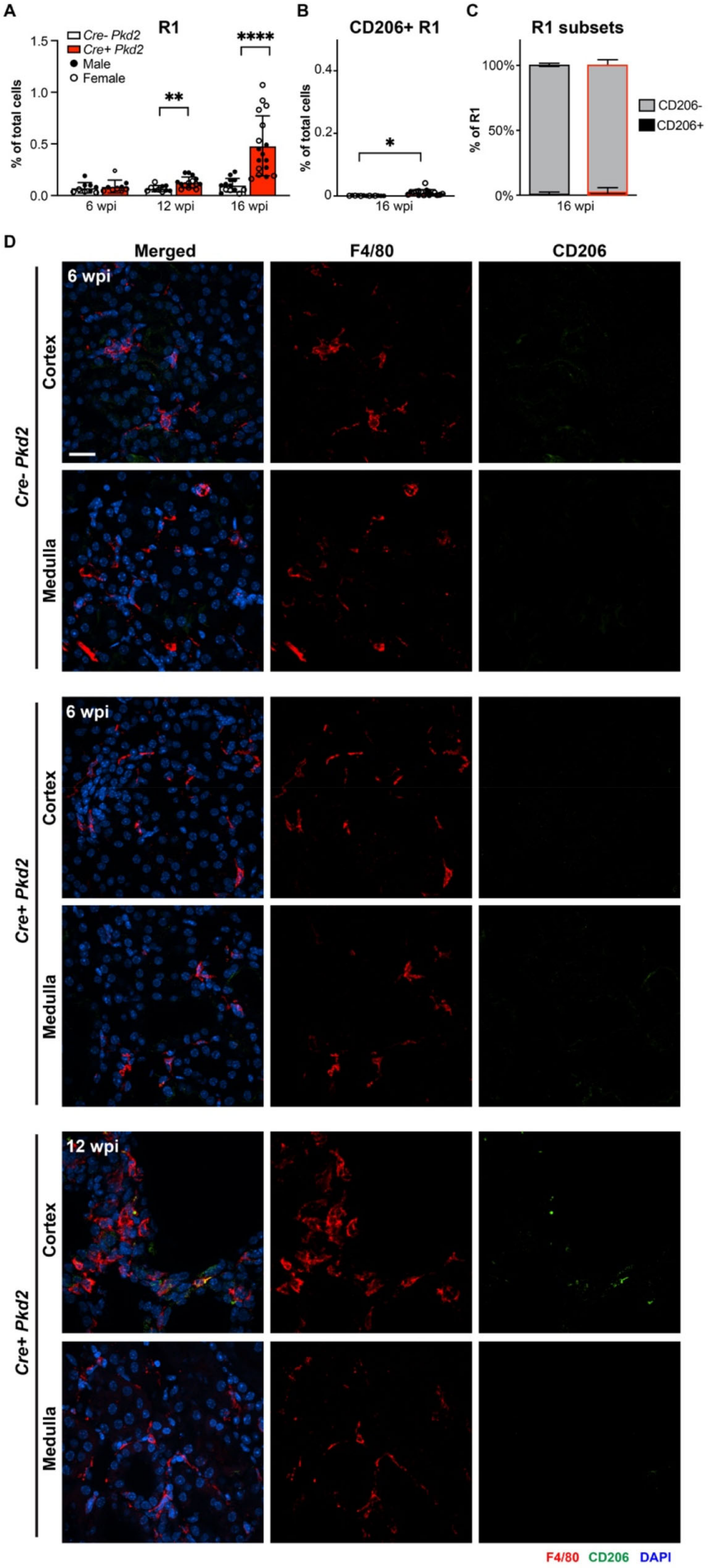

Disease Models & Mechanisms • Supplementary information

Fig. S5. Infiltrating and resident macrophages accumulate in adult-induced *Pkd2* mutant kidneys. (A) The flow cytometry analysis of the percentage of R1 macrophages out of total renal cells at 6-, 12-, 16-week post induction (wpi). The bar graphs showing (B) the percentage of CD206+ R1 out of total cells and (C) the percentage of CD206+ R1 subpopulation out of R1 in *Cre- Pkd2* and *Cre+ Pkd2* at 16 wpi. Each dot represents an individual mouse with solid circles and empty circles representing male and female mice, respectively. Error bars represent ±SD. P<0.05 *, P<0.01 ** and P<0.0001 **** by unpaired t-test. (D) Representative merged and split images showing F4/80 (red), CD206 (green) and DAPI (blue) staining in cortex and medulla for *Cre- Pkd2* and *Cre+ Pkd2* induced at 8 weeks and analyzed at 6- and12- wpi. Scale bar: 20μm.

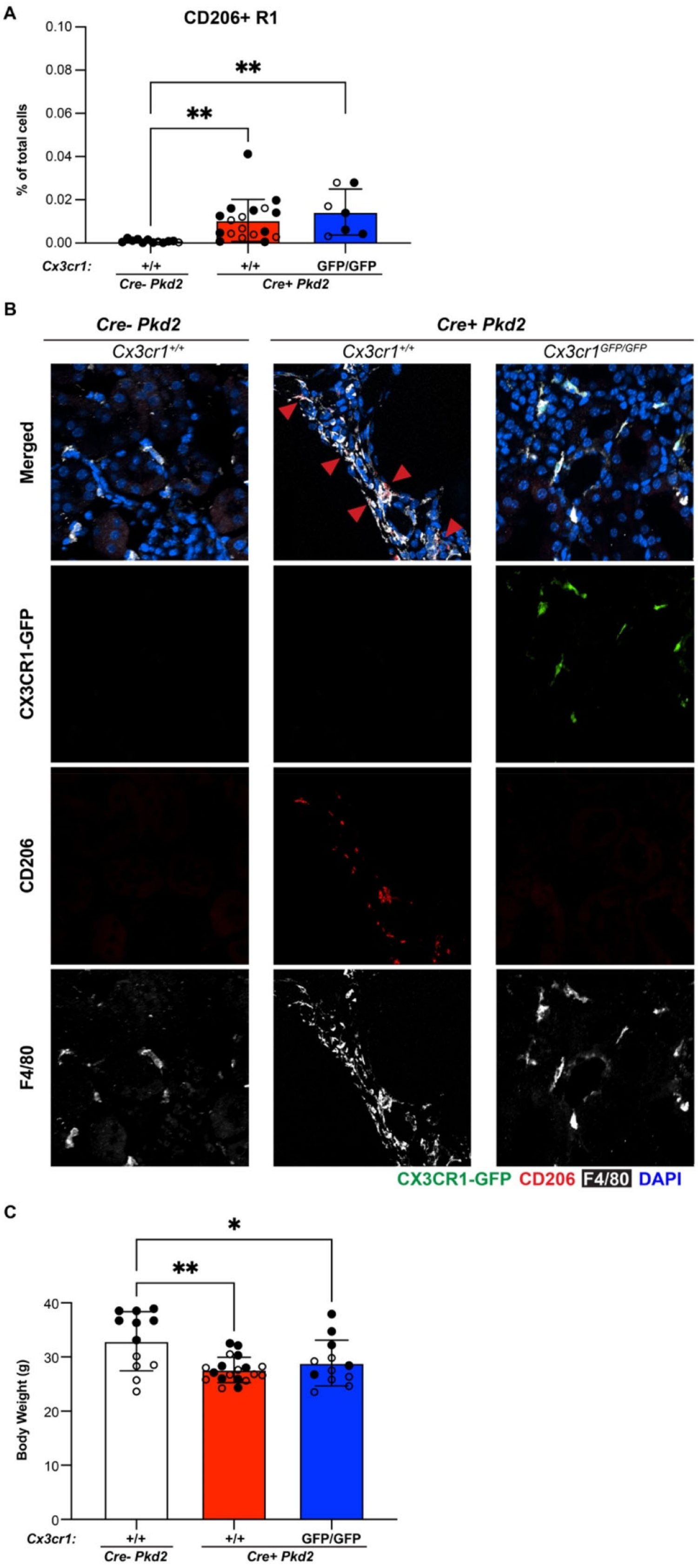

Disease Models & Mechanisms • Supplementary information

Fig. S6. *Cx3cr1* mutation significantly reduces both infiltrating and resident macrophage accumulation in adult-induced *Pkd2* mutant kidneys. (A) Bar graphs showing the percentage of CD206+ R1 out of total cells in adult induced *Pkd2* mutant with *Cx3cr1* control and mutant (*Cx3cr1*$^{GFP/GFP}$) kidneys at 16 wpi. (B) Representative merged and split images showing CX3CR1-GFP positive cells (green), F4/80 (red), CD206 (white) and DAPI (blue) staining in cortex and medulla for each group. Scale bar: 50µm. (C) The bar graphs showing body weight change in each group. Each dot represents an individual mouse with solid circle (male) and empty circle (female). Error bars represent ±SD. P<0.05 * and P<0.01 ** by one-way ANOVA.

Disease Models & Mechanisms • Supplementary information

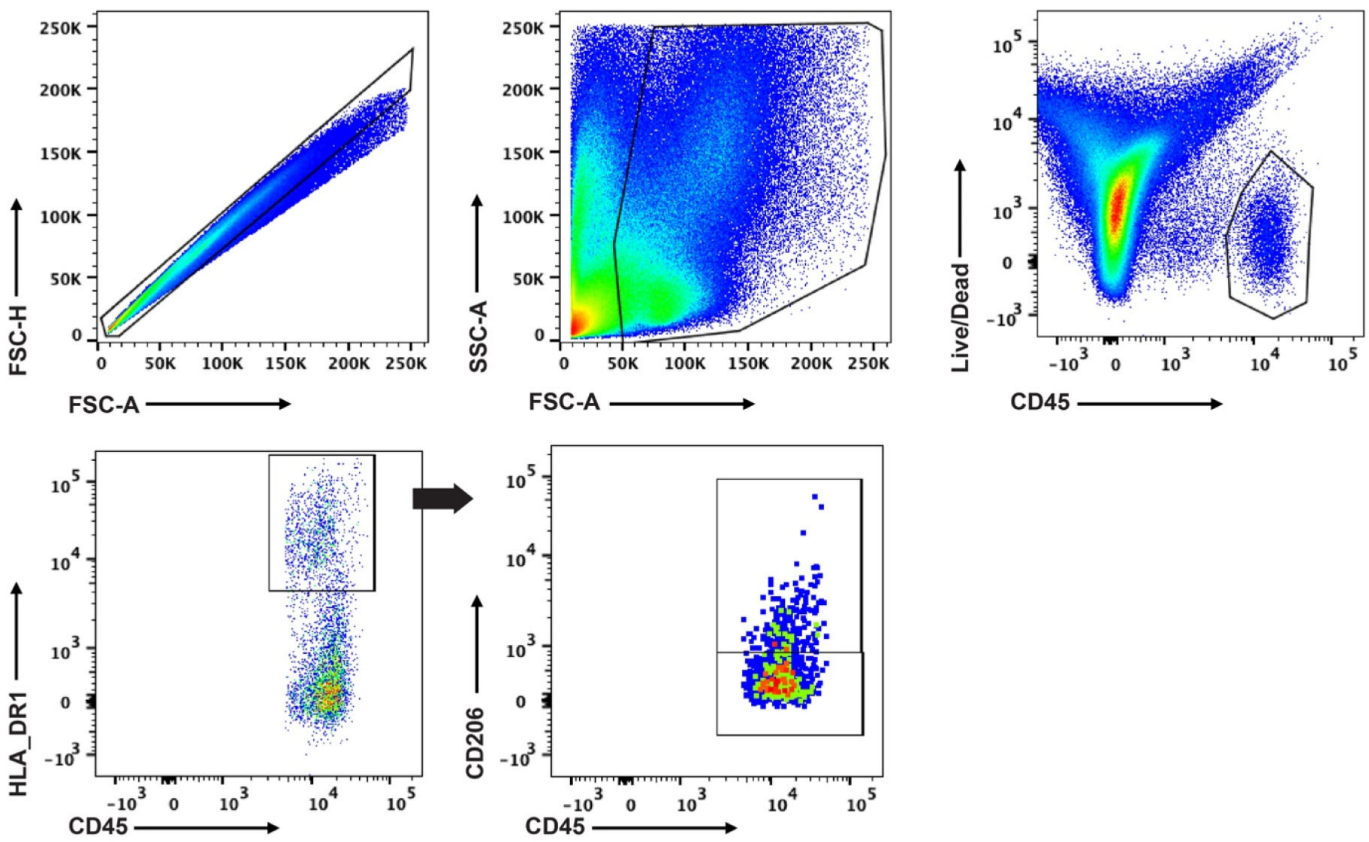

Fig. S7. Gating strategy of flow cytometry analysis used to identify CD206+ macrophages in human kidney tissues. Singlets were gated based on a forward scatter height(FSC-H)/forward scatter area(FSC-A) density plot. Live immunes were identified via the Live/Dead fixable aqua staining and expression of CD45. Antigen-presenting cells were then identified using HLA_DR1 expression. Resulting cells were gated again for detection of the expression of CD206. Those CD206+ cells were defined as CD206+ resident macrophage-like cells based on data from our previous scRNAseq studies showing that CD206 expression is greatly enriched on R2 resident macrophage populations from human kidney (JASN, PMID: 30948627).

Disease Models & Mechanisms • Supplementary information

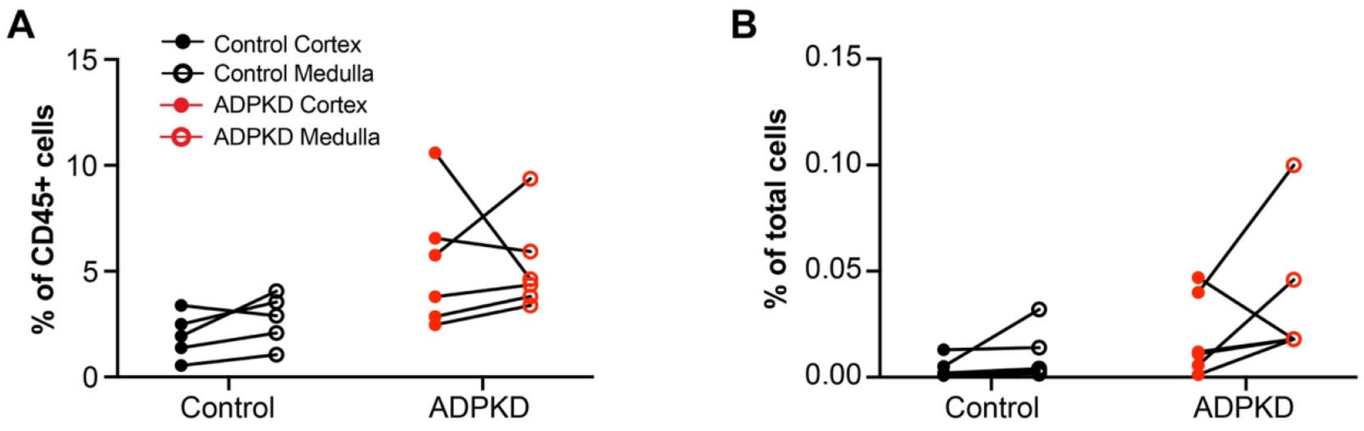

Fig. S8. There is no polarity in macrophage distribution between the medulla vs. cortex. The numbers of CD206+ macrophages in the cortex and medulla of control and ADPKD patients were determined by flow cytometry and are shown (A) as a percentage of CD45+ immune cells and (B) as a percentage of total kidney cells. Black solid circles indicate control cortex, red solid circles indicate ADPKD cortex; black empty circles indicate control medulla, red empty circles indicate ADPKD medulla. No significant differences were identified using a paired t-test comparing medulla vs. cortex values from the same sample with all P values >0.1.

Disease Models & Mechanisms • Supplementary information

Table S1. Clinical characteristics of analyzed kidney tissues

	Control (N=6)	ADPKD (N=7)
**Age (years)**	58±13	53±11
**Gender: male (%)**	67	29
**Race (%)**		
African-American	33	43
Caucasian	67	43
Hispanic	0	14
**eGFR (ml/min/1.73m^2)**	89±31 ***	<10
**Nephrectomy indication (%)**		
Renal cell carcinoma	67	-
Urothelial carcinoma	33	-

Significance: p<0.001 ***

Table S2. List of used antibodies with relevant information

Primary antibody	Host	Provider	Catalog Number	Application
F4/80	Rat	eBioscience	14-4801-82	IF (1:200)
CD206	Rabbit	abcam	ab64693	IF (1:200)
CD45-PE	Rat	eBioscience	12-0451-83	FC for mouse (1:200)
Gr1-APC/Cy7	Rat	BD Bioscience	557661	FC for mouse (1:200)
CD11b-APC	Rat	Invitrogen	17-0112-82	FC for mouse (1:200)
F4/80-eFluor450	Rat	Invitrogen	48-4801-82	FC for mouse (1:200)
CD206-FITC	Rat	BioLegend	141704	FC for mouse (1:200)
Ly-6C PerCP/Cy7	Rat	BD Bioscience	560525	FC for mouse (1:200)
CD45-eFluor450	Mouse	BioLegend	48-9459-42	FC for human (1:200)
HLA_DR-BV650	Mouse	BioLegend	307650	FC for human (1:50)
CD206-BV786	Mouse	BD Bioscience	740999	FC for human (1:100)
**Secondary antibody**	**Host**	**Provider**	**Catalog Number**	**Application**
Anti-Rat-Alexa Fluor 594	Donkey	Invitrogen	A21209	IF (1:1000)
Anti-Rabbit-Alexa Fluor 488	Donkey	Invitrogen	A21206	IF (1:1000)
Anti-Rabbit-Alexa Fluor 647	Donkey	Invitrogen	A21244	IF (1:1000)
DBA-Rhodamine	N/A	Vector	RL-1032-2	IF (1:250)
LTA-FITC	N/A	Vector	FL-1321-2	IF (1:250)

IF: Immunofluorescence; FC: Flow Cytometery

Disease Models & Mechanisms • Supplementary information
